# Metagenome, metatranscriptome, and metaproteome approaches unraveled compositions and functional relationships of microbial communities residing in biogas plants

**DOI:** 10.1007/s00253-018-8976-7

**Published:** 2018-04-30

**Authors:** Julia Hassa, Irena Maus, Sandra Off, Alfred Pühler, Paul Scherer, Michael Klocke, Andreas Schlüter

**Affiliations:** 1 Center for Biotechnology (CeBiTec), Bielefeld University, Genome Research of Industrial Microorganisms, Universitätsstrasse 27, 33615 Bielefeld, Germany; 2 Dept. Biotechnologie, Hochschule für angewandte Wissenschaften (HAW) Hamburg Ulmenliet 20, 21033 Hamburg, Germany; 3 Dept. Bioengineering, Leibniz Institute for Agricultural Engineering and Bioeconomy, Max-Eyth-Allee 100, 14469 Potsdam, Germany

**Keywords:** Anaerobic digestion, Biomass conversion, Methanogenesis, Biogas microbiome, Taxonomic profiling, Genome-enabled metatranscriptomics, Metagenome-assembled genomes, Methanogenic *Archaea*, Sewage digesters, Integrated omics

## Abstract

The production of biogas by anaerobic digestion (AD) of agricultural residues, organic wastes, animal excrements, municipal sludge, and energy crops has a firm place in sustainable energy production and bio-economy strategies. Focusing on the microbial community involved in biomass conversion offers the opportunity to control and engineer the biogas process with the objective to optimize its efficiency. Taxonomic profiling of biogas producing communities by means of high-throughput 16S rRNA gene amplicon sequencing provided high-resolution insights into bacterial and archaeal structures of AD assemblages and their linkages to fed substrates and process parameters. Commonly, the bacterial phyla *Firmicutes* and *Bacteroidetes* appeared to dominate biogas communities in varying abundances depending on the apparent process conditions. Regarding the community of methanogenic *Archaea*, their diversity was mainly affected by the nature and composition of the substrates, availability of nutrients and ammonium/ammonia contents, but not by the temperature. It also appeared that a high proportion of 16S rRNA sequences can only be classified on higher taxonomic ranks indicating that many community members and their participation in AD within functional networks are still unknown. Although cultivation-based approaches to isolate microorganisms from biogas fermentation samples yielded hundreds of novel species and strains, this approach intrinsically is limited to the cultivable fraction of the community. To obtain genome sequence information of non-cultivable biogas community members, metagenome sequencing including assembly and binning strategies was highly valuable. Corresponding research has led to the compilation of hundreds of metagenome-assembled genomes (MAGs) frequently representing novel taxa whose metabolism and lifestyle could be reconstructed based on nucleotide sequence information. In contrast to metagenome analyses revealing the genetic potential of microbial communities, metatranscriptome sequencing provided insights into the metabolically active community. Taking advantage of genome sequence information, transcriptional activities were evaluated considering the microorganism’s genetic background. Metaproteome studies uncovered enzyme profiles expressed by biogas community members. Enzymes involved in cellulose and hemicellulose decomposition and utilization of other complex biopolymers were identified. Future studies on biogas functional microbial networks will increasingly involve integrated multi-omics analyses evaluating metagenome, transcriptome, proteome, and metabolome datasets.

**Electronic supplementary material:**

The online version of this article (10.1007/s00253-018-8976-7) contains supplementary material, which is available to authorized users.

## Introduction

In Europe, flammable swamp gas is presumably known since the roman period. Microbial anaerobic digestion (AD) of plant material as origin of burnable biogas was scientifically recognized and analyzed in detail since mid of the twentieth century. Complex microbial consortia are responsible for successive degradation of organic biomass to biogas consisting of methane (CH_4_) and carbon dioxide (CO_2_) and, in smaller proportions, of other gases. In industrial-scale biogas plants (BGPs), biogas is produced by AD from agriculturally produced renewable resources such as maize, grass, and sugar beet, and even biodegradable organic wastes can be used as substrates (Weiland 2010; Zhang et al. 2016). Today, biogas production considerably contributes to the recovery of energy from renewable resources thereby also positively affecting the balance of the climate-relevant gas carbon dioxide. In Germany, nearly 9000 biogas plants (including about 450 bio-waste biogas plants), with 4.5 Giga-Watt installed electric power and about 1 Giga-Watt thermal energy usage, are operated (FNR 2017).

The biomethanation process is formally subdivided into four phases, i.e., (i) hydrolysis/cellulolysis of complex organic compounds, namely carbohydrates, proteins and lipids, towards corresponding oligomers and monomers, (ii) acidogenesis (fermentation) of the latter metabolites to the intermediates propionate, butyrate, other short-chain volatile fatty acids (VFA) and alcohols, (iii) acetogenesis of primary fermentation products to acetic acid, CO_2_, and H_2_, and (iv) methanogenesis resulting in CH_4_ and CO_2_ (Angenent et al. 2004). The first three phases are solely performed by fermentative Bacteria. Only certain methanogenic *Archaea* are able to synthesize CH_4_ from the end-products of bacterial fermentation. Despite the technical improvements of anaerobic wastewater treatment in the beginning and mid of the twentieth century, for a long period, anaerobic and biomass degrading microorganisms were regarded as a “relatively unimportant group of organisms” (McBee 1950). However, their potential for the development of economically valuable bioconversion processes utilizing cellulosic or organic waste materials for production of valuable end-products was recognized.

First attempts to study microbial biogas communities relied on cultivation-based approaches and started in the beginning of the twentieth century (e.g., Schnellen 1947; McBee 1954) resulting in over 150 newly discovered species of microorganisms (Söhngen et al. 2016). Technical improvements concerning anaerobic cultivation of microorganisms are still leading to the isolation and characterization of new type strains for hydrolytic/acidogenic *Bacteria* such as *Clostridium bornimense*, *Herbinix hemicellulosilytica*, *Herbinix luporum*, *Herbivorax saccincola, Proteiniphilum saccharofermentans*, *Petrimonas mucosa*, *Fermentimonas caenicola,* and *Proteiniborus indolifex* (Hahnke et al. 2014; Koeck et al. 2015; Hahnke et al. 2016; Koeck et al. 2016a; Koeck et al. 2016b; Hahnke et al. 2018). Likewise, new species for methanogenic *Archaea* such as *Methanobacterium aggregans* or *Methanosarcina flavescens* were described (Kern et al. 2015, 2016). However, the options of cultivation-based approaches to uncover all members of biogas communities are intrinsically highly limited. Thus, cultivation-independent methods are indispensable to tackle the whole complexity of biogas communities.

Metagenomics gained in importance for the dissection of microbial assemblages in the same way as the performance and efficiency of next-generation sequencing (NGS) technologies were advanced. Inevitable, anaerobic digestion communities were elucidated by applying methods of metagenome, genome and post-genome research taking advantage of high-throughput sequencing of environmental whole community DNA and RNA. In the present review, state-of-the-art metagenomics approaches are presented to illustrate their usefulness in analyses tackling microbial communities residing in BGPs. To elucidate the metabolically active biogas community, metatranscriptome, metaproteome, and metabolome studies are addressed in an integrated manner.

It is commonly accepted that biogas producing microbial communities are the key for process shaping and development of optimization strategies since they provide opportunities for their management and engineering (Carballa et al. 2015; Koch et al. 2014). Accordingly, this article reviews current knowledge on structure and performance of microbial communities residing in full-scale biogas-producing reactors considering microbiome management and monitoring options. Previous reviews addressing microbial communities of anaerobic digestion systems were mainly focused on laboratory-scaled reactors and classical molecular methods to uncover community compositions and functions (e.g., Demirel and Scherer 2008; Nasir et al. 2012; Čater et al. 2013; Venkiteshwaran et al. 2015; Schnürer 2016; Demirel 2014; Vanwonterghem et al. 2014). In the present review, microbial communities of BGPs converting agriculturally produced renewable primary products, organic residues, and manure were considered. Laboratory systems were only included if they feature pilot character exemplifying fundamental methodological approaches or important insights in microbial community structure and functionality.

## Taxonomic characterization of microbial communities by high-throughput 16S rRNA gene amplicon sequencing

To obtain direct and immediate insights into microbial community compositions and the phylogenetic relationship of community members, specific marker genes were studied by their PCR-amplification from whole community (metagenomic) DNA. A widely and commonly used approach for microbial community profiling without prior cultivation is the analysis of the 16S small subunit ribosomal-RNA (rRNA) gene sequence (Woese 1987; Lebuhn et al. 2014; Simó et al. 2014). As an integral part of the ribosome, this rRNA is ubiquitously present in prokaryotic organisms. With a size of about 1500 bp, the 16S rRNA gene comprises nine hypervariable regions (V1-V9) separated by conserved regions. The hypervariable regions provide organism specific sequences enabling identification of taxa. The whole 16S rRNA gene or specific regions can be considered as taxonomic marker gene (Yang et al. 2016). Currently, as taxonomic thresholds for the species level, 98.65% and for the genus level, 94.5% 16S rRNA gene nucleotide sequence identity are proposed (Kim et al. 2014; Yarza et al. 2014).

To study the highly diverse and dynamic microbial community structures present in environmental systems and to minimize the costs for DNA sequencing, initially, several fingerprinting techniques targeting the 16S rRNA gene were developed such as denaturing gradient gel electrophoresis (DGGE) analysis (Muyzer et al. 1993), amplified ribosomal DNA restriction analysis (ARDRA) of 16S rRNA gene libraries (Moyer et al. 1994), and terminal restriction fragment length polymorphisms (T-RFLP) analysis (Liu et al. 1997). These approaches were applied in previous studies on microbial structures present in anaerobic digesters and biogas reactors (Cabezas et al. 2015), as examples, DGGE for the analysis of mesophilic anaerobic digestion of municipal waste (Silvey et al. 2000), ARDRA for the analysis of mesophilic methanogenic bioreactors supplied with artificial media (Fernandez et al. 2000), and ARDRA combined with T-RFLP for the analysis of psychrophilic anaerobic digestion of synthetic industrial wastewaters (Collins et al. 2003).

The advantage of DGGE and ARDRA as low-cost methods providing rapid insights into community structures and dynamics resulted in their continued application*,* e.g., to study the impact of the addition of cellulolytic/hydrolytic enzymes to anaerobic digestion (Wang et al. 2016) or to follow microbial community dynamics in a BGP (Yamei et al. 2017). Also semi-automated, computer-aided T-RFLP analysis still is a valuable tool to study the development of microbial populations depending on changes of technical process parameters. As examples, Goux et al. (2015) analyzed the response of microbial communities on stress factors in laboratory scaled experiments. Krakat et al. (2010), Argyropoulos et al. (2013), and Theuerl et al. (2015) showed that even the microbiome of a well-running BGP undergoes structural fluctuations, and Witarsa et al. (2016) characterized the influence of different starter *inocula*.

However, due to the rapidly decreasing costs for high-throughput DNA sequencing, fingerprinting tools for monitoring of microbial community structures are now commonly applied in combination with 16S rRNA gene targeted amplicon NGS, e.g., DGGE together with Ion Torrent sequencing (Akyol et al. 2016), or T-RFLP together with 454-pyrosequencing or Illumina sequencing (Goux et al. 2015; Sun et al. 2016; Liu et al. 2017; Ozbayram et al. 2017; Ziganshina et al. 2017). Fingerprinting techniques allow the rapid pre-screening of samples for NGS, or the focusing on particular functional groups of microorganisms, e.g., by targeting glycoside hydrolase genes to access cellulose-degrading *Bacteria* (Sun et al. 2016) or the genes for the methyl-coenzyme-M-reductase to monitor exclusively methanogenic *Archaea* (Ozbayram et al. 2017).

**Fig. 1:**
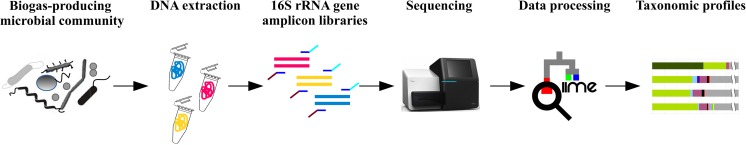
Schematic overview on taxonomic profiling of biogas-producing microbial communities applying 16S rRNA gene amplicon sequencing. After extraction of whole community DNA, 16S rRNA gene amplicon libraries were constructed and subsequently sequenced. Obtained sequences were processed with the program QIIME (Caporaso et al., 2010) to calculate taxonomic community profiles

The development of NGS platforms led to an optimized 16S rRNA gene analysis, because they enabled direct sequencing of DNA libraries and thus several hundreds of samples can be sequenced in parallel (see Fig. 1). NGS platforms usually generate short read lengths. Therefore, only a single or a combination of adjacent hypervariable 16S rRNA gene regions can be sequenced. It was shown that despite these short read lengths, a sufficiently accurate taxonomic classification of microbial communities can be achieved (Liu et al. 2008). The 16S rRNA gene fragments can be amplified by PCR applying universal primers (e.g., Takahashi et al. 2014) or bacterial and archaeal specific primers. In this context, amplification of different hypervariable regions of the 16S rRNA gene and application of different sequencing technologies used for taxonomic community profiling should be considered carefully since both factors have a strong impact on the obtained results as shown previously by Tremblay et al. (2015) analyzing a mock community composed of a known number of species. Moreover, an essential step after sequencing of constructed amplicon libraries is the quality control which ensures increased accuracy and prevents overestimation of community diversity. Pre-processing of amplicon datasets involves several steps (Jünemann et al. 2017) including removal of chimeric sequences. Subsequently, sequences are subjected to operational taxonomic units (OTUs) clustering, where usually a cutoff of 97% sequence similarity is used, taxonomic classification, and statistical analysis. For reference-based taxonomic classification, 16S rRNA gene reference databases like SILVA (Quast et al. 2013) and RDP (Cole and Tiedje 2014) are consulted. For further analyses of sequence datasets such as rarefaction estimations, calculation of diversity metrics, principle component analyses (PCA), or calculation of UniFrac distances, different bioinformatics tools are available (Schloss et al. 2009; Caporaso et al. 2010). Despite the benefits of taxonomic community profiling based on 16S rRNA gene sequencing, this method faces some limitations. The PCR-based amplification of the target region is biased due to primer properties. Moreover, the resolution of the method is limited due to short read lengths and may lead to underestimation of species diversity. Since the 16S rRNA gene copy numbers vary for different species, taxa abundance estimations may be biased (Větrovský and Baldrian 2013). This problem can be addressed bioinformatically, provided that the gene copy numbers of the species involved are known which frequently is not the case for unknown microbial communities. Moreover, 16S rRNA gene amplicon analyses depend on completeness and correctness of corresponding reference databases for sequence classification (Ranjan et al. 2016). However, the 16S rRNA gene amplicon sequencing method has often been used for characterizing microbial communities in biogas plants because of its advantageous cost-benefit relation. To compensate for resolution biases (like underestimation of species diversity) associated with sequencing of individual variable regions, the approach of “full-length” 16S rRNA amplicon sequencing by means of the PacBio© single molecule, real-time (SMRT) technology can be considered (Wagner et al. 2016).

### Taxonomic composition of bacterial communities residing in biogas plants

Microbial communities of biogas fermenter samples consist of bacterial and archaeal sub-communities. These differ in their diversity and abundances depending on the BGP operating parameters such as temperature, fed substrates, pH, and reactor and fermentation type (Weiland 2010; Yu et al. 2014; Abendroth et al. 2015). For several BGPs operated under mesophilic (35–45 °C) or thermophilic (45–60 °C) conditions, fed with different substrates like agricultural residues, manure, and/or sewage sludge, the community composition was analyzed. It was reported that relative abundances of the bacterial community within these fermenters amount from 80 to 100% (Sundberg et al. 2013; Maus et al. 2017). Remaining portions are mainly occupied by methanogenic *Archaea* (see chapter 3.2.). While the four phases of AD and their principal pathways are known, the exact taxonomic compositions and network dynamics of corresponding communities are still only partly understood. Since 16S rRNA gene amplicon sequencing is convenient to provide insights into the taxonomic composition of complex microbial communities, it has often been the method of choice for a number of studies on the correlation of process parameters and/or environmental conditions and community structure (refer to chapter 2.).

**Fig. 2:**
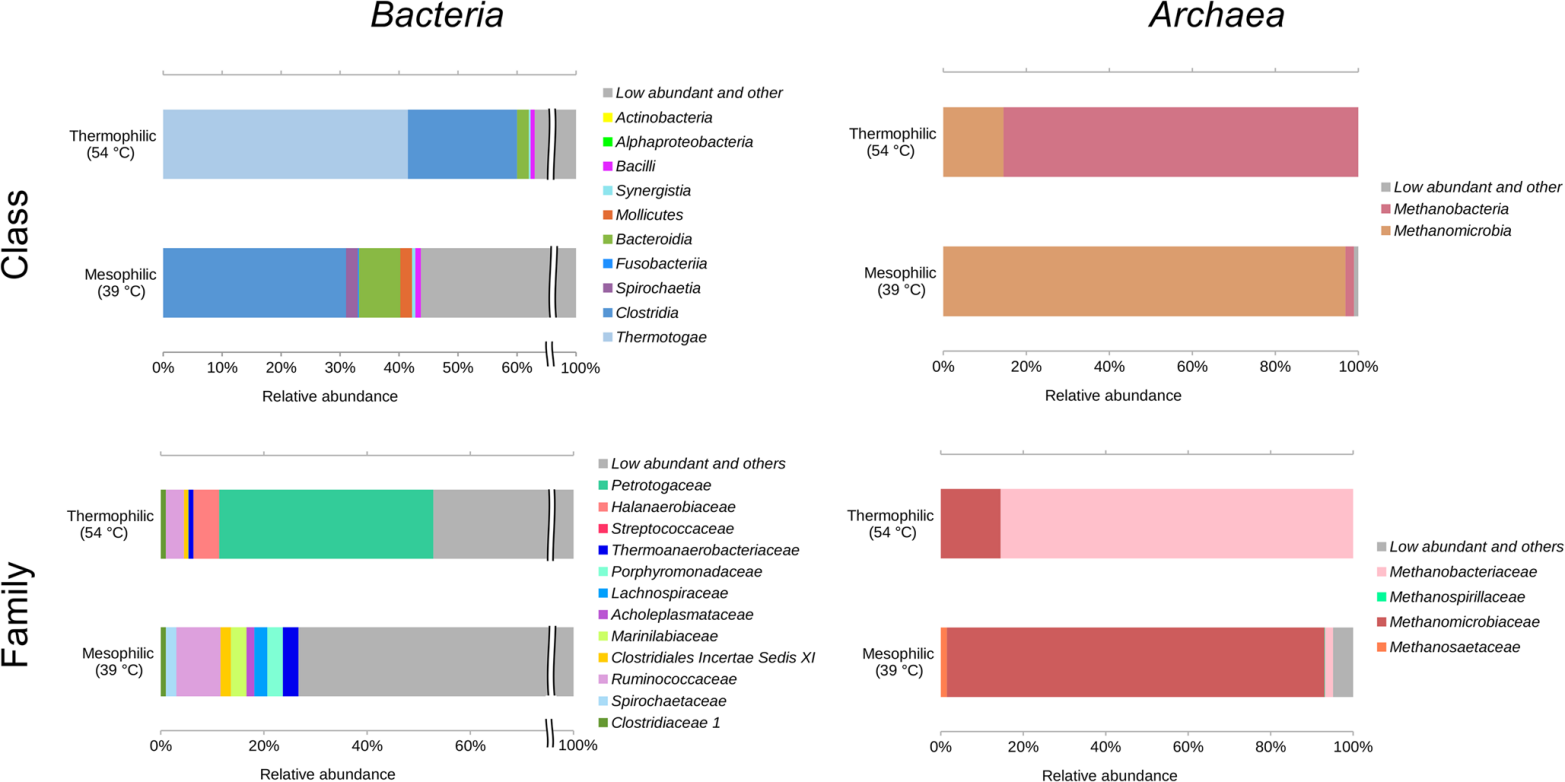
Taxonomic profiling of microbial communities residing in exemplary mesophilic and thermophilic biogas plants fed with agricultural (by-) products based on 16S rRNA gene amplicon sequencing (Maus et al. 2016b). For amplicon processing, a pipeline including FLASH (Magoč and Salzberg 2011), UPARSE (Edgar 2013), Usearch 8.0 (Edgar 2010), and RDP classifier (Wang et al. 2007) was used as described recently by Maus et al. (2016b). Relative abundances of the most abundant classes and families of bacterial (left) and archaeal (right) communities were shown

By these approaches, dominant and therefore important taxa of the biogas formation process were identified. Regarding the bacterial part of the community, the majority of relevant studies described predominance of the bacterial phyla *Firmicutes* and, under certain conditions, of *Bacteroidetes* and *Thermotogae*. An example of the mesophilic and thermophilic bacterial community composition is shown in Fig. 2 (Maus et al. 2016b). These taxa are believed to belong to the core microbiome of biogas-producing microbial communities (e.g., Rui et al. 2015). However, the ratio of these phyla is very much related to the respective temperature, fed substrates, and process conditions (Sundberg et al. 2013; Rui et al. 2015). Furthermore, representatives belonging to other phyla such as *Proteobacteria*, *Spirochaetes*, *Tenericutes, Verrucomicrobia, candidate phylum Cloacimonetes* (previously named WWE1)*, Acidobacteria*, and *Chloroflexi* have prevalently been detected in mesophilic reactors, but typically at comparatively lower abundances (Klocke et al. 2007; Sundberg et al. 2013; Ziganshin et al. 2013; St-Pierre and Wright 2014; Rui et al. 2015; Li et al. 2013; Sun et al. 2016; Stolze et al. 2016).

In mesophilic full-scale reactors fed with maize silage and manure as substrates, the classes *Clostridia* and *Bacilli* were highly abundant within the phylum *Firmicutes* (Stolze et al. 2016; Treu et al. 2016a). In particular, many species belonging to the genus *Clostridium* are involved in decomposition of complex carbohydrates including cellulose, xylan, amylose, and amylopectin and represent hydrolysis key players (Labbe and Duncan 1975; Dürre 2005). Some mesophilic *Clostridia* species such as *Acetoanaerobium sticklandii* (Fonknechten et al. 2010) and *Butyrivibrio proteoclasticus* (Attwood et al. 1996) are able to degrade proteins in addition to complex carbohydrates (Schnürer 2016; Li et al. 2013).

The class *Clostridia* also comprises species capable of performing acetogenesis as well as syntrophic fatty acid degradation. Corresponding syntrophs belong to the families *Thermoanaerobacteriaceae*, *Costridiaceae*, and *Syntrophomonadaceae* (Schnürer 2016). In addition, the phylum *Proteobacteria* also includes many syntrophs belonging to the genera *Syntrophus*, *Pelobacter*, *Smithella*, *Syntrophorhabdus*, and *Syntrophobacter* described to live in association with methanogenic *Archaea* (Jackson et al. 1999; Bok et al. 2001; Qiu et al. 2008).

Mesophilic digesters fed with protein-rich and hardly digestible substrates such as straw showed prevalence of the families *Bacteroidetes, Porphyromonadaceae*, and *Marinilabiaceae* (Sun et al. 2015; Moset et al. 2015). *Bacteroidetes* representatives are known to ferment sugars to acetate and propionate. Some studies reported on higher abundances of *Porphyromonadaceae* members in reactors operating at, e.g., high organic loading rates (OLRs) or nitrogen/ammonia levels caused by high protein content of the used substrates (Goux et al. 2015; Müller et al. 2016). This observation led to the suggestion that these may serve as potential marker microorganism for deteriorated biogas process conditions. In general, despite the overall dominance of a few taxa, a high degree of variation is often seen within mesophilic bacterial communities, driven by the composition of the fed substrate, ammonia levels, and by the operating conditions applied.

In thermophilic BGPs, members of the phylum *Firmicutes* also dominate the bacterial community followed by *Thermotogae* and *Bacteroidetes*. Members of the class *Clostridia* were more abundant in thermophilic compared to mesophilic digesters. One of the most prominent representatives of the genus *Clostridium* is *C. thermocellum*, which is known to be a very efficient thermophilic cellulose degrader (Akinosho et al. 2014; Koeck et al. 2014). Likewise, the families *Lachnospiraceae* and *Halanaerobiaceae* are prevalent under thermophilic conditions (Maus et al. 2016b; Stolze et al. 2016). Cellulolytic *Lachnospiraceae* species are mainly responsible for the degradation of complex plant material. For example, *Herbinix hemicellulosilytica* T3/55^T^ isolated from a thermophilic full-scale BGP (Koeck et al. 2015) is able to digest cellulose. Likewise, *Halocella cellulolytica* (*Halanaerobiaceae*) was described to degrade cellulose producing acetate, ethanol, lactate, H_2_, and CO_2_ as end-products. Moreover, *Halocella* spp. are capable to tolerate high salt concentrations frequently occurring in biogas reactor environments (Simankova et al. 1993).

In recent years, the taxon *Thermotogae* was recognized to be of importance for the biogas-production process. In this class, the genera *Defluviitoga* (Ben Hania et al. 2012) and *Petrotoga* (Lien et al. 1998) are determined as predominant genera in particular BGPs (Maus et al. 2016a; Stolze et al. 2016). Corresponding species utilize a large variety of saccharides (Maus et al. 2016a; Lien et al. 1998) for acetate, ethanol, CO_2_, and H_2_ production.

However, it should be noted that BGP microbiomes still comprise a high diversity of uncharacterized *Bacteria* (from 14 to 41%, Stolze et al. 2015; Maus et al. 2016b) such as the candidate taxa Hyd24–12 (Kirkegaard et al. 2016), OD1 (Peura et al. 2012), TM7 (also known as candidatus *Saccharibacteria*) (Ferrari et al. 2014), and SR1 (Harris et al. 2004), often only known by their 16S rRNA gene sequence. Clarifying the ecological role of uncultured *Bacteria* still is addressed in ongoing research.

### Taxonomic composition of archaeal communities residing in biogas plants

To evaluate the occurrence of methanogenic *Archaea* in biogas communities, 78 full-scale anaerobic digesters described in 17 recent publications (2008–2017) were considered. The main attributes and features of identified archaeal biogas community members are summarized in Table 1. The comparison of the process parameters of BGPs with respect to the observed dominant genera of methanogens shows that apparently fed substrates affect the microbial community more than temperature or hydraulic retention time (Cardinali-Rezende et al. 2012; Franke-Whittle et al. 2014; Han et al. 2017; Lee et al. 2014; Lucas et al. 2015; Nettmann et al. 2008; St-Pierre and Wright 2013; Zhu et al. 2011). Different substrates account for different ammonium/ammonia contents in the fermentation sludge which significantly affect the composition of the microbial community (Fotidis et al. 2014). Moreover, the amount and type of nutrients and minerals present in the substrate also shape the methanogenic sub-community (Fontana et al. 2016; Luo et al. 2016). Previous reviews indicated a dominance of the hydrogenotrophic pathway in anaerobic digesters with a high nutrient content like BGPs fed with agricultural material or municipal bio-waste (Demirel and Scherer 2008; Demirel 2014). An increased hydrogen partial pressure like under thermophilic conditions may favor hydrogenotrophic methanogens for thermodynamic reasons (Zinder 1990).

**Table 1:** Summarized data of 78 mesophilic and thermophilic BGPs (as referenced in Supplemental Table S1) regarding predominant methanogenic genera, type of biogas plant, temperature, hydraulic retention time, and ammonia content

Predominant methanogenic genus	Type of BGP/substrate	Temperature (°C)	HRT (days)	Ammonia (NH_4_^+^-N) (g L^−1^)
*Methanosaeta*	Municipal sewage sludge	35–43 (51–53)	10–30	< 1.3
	Different agricultural by-products	39–43	> 32	< 1.9
*Methanoculleus*	Different agricultural by-products	35–45 and 50–62	3–108	1.4–5.4
*Methanosarcina*	Different agricultural by-products	35–45 and 52–53	21–138	1.8–2.7
*Methanobrevibacter*	Anaerobic digestion of feces (animal manure or organic fraction of municipal solid waste)	37–42 (51–53)	20–150	0.7–4.0
*Methanobacterium*	Different agricultural by-products and organic fraction of municipal solid waste	Mesophilic and 50–55	20–80	1.3–2.2
*Methanothermobacter*	Different agricultural by-products	> 55	16–26	2.9–3.8
*Methanomethylovorans*	Municipal and industrial sewage sludge	Mesophilic	33	n.d.

Ammonia (NH_3_) is in balance with ammonium (NH_4_^+^) according to pH (mainly about 8.0 in BGPs) and temperature

*BGP* biogas plant, *HRT* hydraulic retention time, *n.d.* no data

**Fig. 3:**
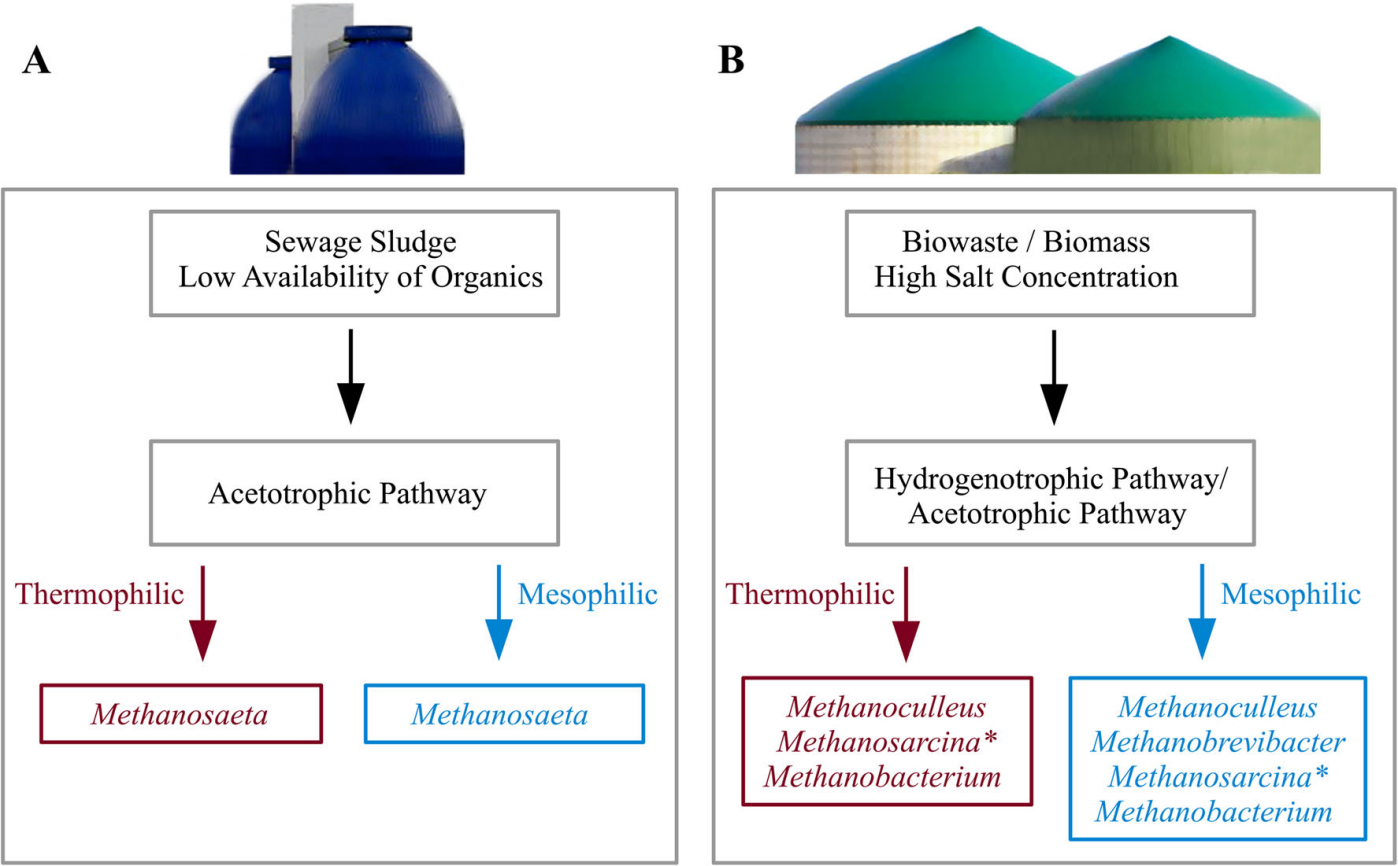
The predominant archaeal methanogenic genera as summarized by recent literature covering 78 mesophilic and thermophilic BGPs (as referenced in Supplemental Table S1). *Methanosarcina mainly occurred as single cells instead of typical aggregates (single-coccoid form, ≤ 1 μm)

In municipal sewage digesters, consistently the acetoclastic genus *Methanosaeta* dominated (see Fig. 3). Due to its high substrate affinity with a K_S_–value of 0.4 to 1.2 mM (Jetten et al. 1990), it outcompetes other methanogens under low acetate concentrations. Digested sewage sludges are known to consist of generally low amounts of easily degradable compounds, but represent high substrate diversity. This coincides with pioneering investigations by isotopic methods (Kaspar and Wuhrmann 1978). Additionally, a correlation between high concentrations of total ammonia (sum of NH_3_ and NH_4_^+^) and the absence of *Methanosaeta* in biogas reactors was observed (Karakashev et al. 2005; Nettmann et al. 2010).

Usually, agricultural BGPs are operated under mesophilic conditions (35–45 °C). In contrast, thermophilic full-scale plants are managed at temperatures of approx. 50 to 55 °C. BGPs of the agricultural type are characterized mainly by high salt (10–20 g KCl equivalent per liter) and high ammonium concentrations. They were dominated by the genera *Methanoculleus*, *Methanosarcina*, and *Methanobrevibacter*. All three genera included species adapted to either thermophilic or mesophilic temperatures.

At high ammonia levels (> 2.8 g L^−1^ NH_4_^+^-N), methane production in BGPs generally occurs through syntrophic acetate oxidation and hydrogenotrophic methanogenesis (Ek et al. 2011; Fotidis et al. 2014). This suggests that hydrogenotrophic methanogenic *Archaea* belonging to *Methanomicrobiales* spp. and *Methanobacteriales* spp. are non-susceptible to ammonia toxicity. Prevalent occurrence of the order *Methanomicrobiales* could also be modulated by a high hydrogen affinity. Corresponding species exhibit a low threshold concentration for hydrogen of about 0.1 μM, respective 15 Pa H_2_-pressure (Lee and Zinder 1988), possibly providing an advantage over certain members of the order *Methanobacteriales*.

The genus *Methanoculleus* has previously been reported to be related to relatively elevated ammonium levels (Schnürer et al. 1999; Schnürer and Nordberg 2008; Nettmann et al. 2010; Ziganshin et al. 2013). Beside the tolerance towards ammonia (1.4–5.4 g L^−1^ NH_4_^+^-N, Table 1), *Methanoculleus* appears to be highly adaptable to process parameters such as temperature and hydraulic retention time with 35–45 or 50–62 °C and 3–108 days, respectively (see Table 1). Therefore, the reason why *Methanosarcina* dominates over *Methanoculleus* in some BGPs, which mainly ferment different types of manure and agricultural by-products, cannot be directly correlated to one of the different process parameters listed in the Supplemental Table S1. However, it is well-known that *Methanosarcina* sp. are robust towards different detrimental conditions such as high ammonia or salt concentrations, or fluctuating operational conditions such as temperature shifts (De Vrieze et al. 2012). The third most predominant methanogenic genus is *Methanobrevibacter*. It prevails compared to other genera, especially in the fermentation of fecal matter in mesophilic biogas reactors (Table 1; Supplemental Table S1). The genera *Methanobacterium* and *Methanothermobacter* often seem to be accompanied by a second abundant methanogen in thermophilic BGPs fed with variable agricultural by-products and the organic fraction of municipal solid waste including fat-rich waste (Supplemental Table S1).

The only example for a predominant methanogen which is involved in the methylotrophic pathway (Deppenmeier et al. 1996) is the genus *Methanomethylovorans*. It was found to be highly abundant together with *Methanosaeta* in a mesophilic anaerobic digestion reactor fed with municipal and industrial sewage sludge. The analyzed reactor featured a very low OLR (0.5 kg VS m^−3^ d^−1^) (Table 1; Supplemental Table S1). However, the abundance of *Methanomethylovorans* might be connected to the presence of particularly high amounts of oil and alcohols such as methanol, since the analyzed digester was supplemented with remnants from biodiesel production.

Regarding the percentages of the dominant genera, niche formation is noticeable in case of changing substrates. For example, over 90% of the archaeal community is dominated by one genus when substrates such as food-waste-recycling wastewater (FRW), the organic fraction of municipal solid waste (OFMSW) or slaughterhouse waste (SHW) were fed. Only in two biogas plants, the archaeal sub-community is composed of only two genera (see Supplemental Table S1).

In terms of diversity, Kirkegaard et al. (2017) showed that the archaeal community of thermophilic samples among 32 sewage sludge digesters of 20 wastewater treatment plants featured a lower diversity than the mesophilic samples (Kirkegaard et al. 2017). In contrast, other studies revealed that the impact of the temperature on the archaeal diversity was weaker compared to that of the substrates, and their conditional nutrient and ammonium contents (Sundberg et al. 2013; Luo et al. 2016). While the sewage sludge digesters revealed a diversity of up to 16 different methanogenic genera (6–16, on average 11, *n* = 30 full-scale digesters, 24 under mesophilic [34–40 °C] and six under thermophilic conditions [51–55 °C]), the co-digesters (agricultural by-products, organic fraction of municipal solid waste, slaughterhouse waste) achieved a diversity of up to 8 different methanogenic genera (1–8, on average 4, *n* = 20 full-scale BGPs, 11 under mesophilic [37–40 °C] and nine under thermophilic conditions [50–55 °C]) (Sundberg et al. 2013; Luo et al. 2016; Kirkegaard et al. 2017).

## The genomic potential of communities in biogas plants by sequencing of community DNA

**Fig. 4:**
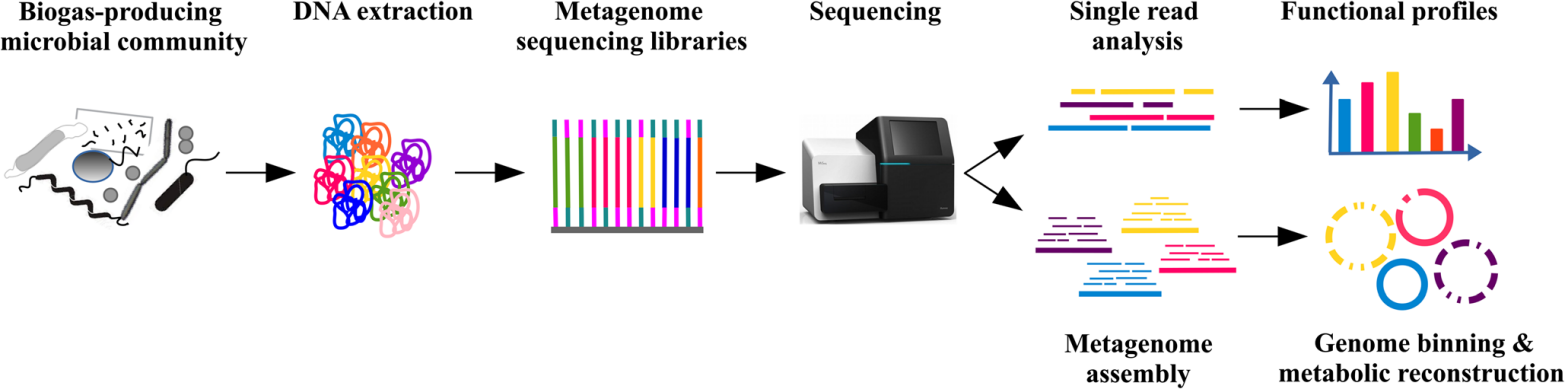
Workflow for functional profiling of microbial biogas communities exploiting metagenome sequence data. After sampling at biogas reactors, total DNA was extracted for construction of whole metagenome shotgun libraries which were subsequently sequenced on high-throughput sequencing platforms. Resulting sequencing data were quality checked and functionally characterized based on single read sequences in order to deduce functional profiles of the underlying biogas community. Moreover, metagenome assembly followed by a binning approach was applied to compile MAGs, which were then analyzed for their metabolic potential

NGS 16S rRNA gene amplicon sequencing enabled high-resolution taxonomic profiling of biogas-producing microbial communities. However, to gain insights into their functional potential, sequencing of the whole metagenome is indispensable. Analysis of metagenome sequence data can be done in either of two possible ways: (i) functional information can be assigned to single metagenome sequence reads regarding them as Environmental Gene Tags (EGTs) (Krause et al. 2008); and (ii) metagenome assembly and binning strategies were followed to gain deeper insights into genome sequence information of so far non-cultivable biogas community members (see Fig. 4).

### The genomic potential of microbial communities by single-read metagenomics

Different approaches are applied to assign functional information to single metagenome reads (see Fig. 4). Common tools for this task are for example MG-RAST (Meyer et al. 2008), MEGAN 6 (Huson et al. 2016), or eggNOG (Huerta-Cepas et al. 2016). Implemented bioinformatics pipelines usually comprise one or a combination of different functional classification systems referring to COG (cluster of orthologous groups) categories (Tatusov et al. 2000), SEED subsystems (Overbeek et al. 2014), eggNOG orthologous groups (Huerta-Cepas et al. 2016), or InterPro entries (Finn et al. 2017) which includes different protein signature databases (e.g., Pfam). The reconstruction of metabolic pathways of the anaerobic digestion process can be done by using databases like KEGG (Kyoto Encyclopedia of Genes and Genomes; Kanehisa et al. 2017), MetaCyc (Caspi et al. 2016), and BRENDA (Chang et al. 2009). Potential functions of the microbial community as well as abundances of involved genes are determined by assigning metagenome reads (e.g., by BLASTX) to defined pathways (Cai et al. 2016).

Functional classification according to COG categories of a metagenome from a mesophilic full-scale BGP fed with renewable primary products was firstly applied by Schlüter et al. (2008). The major identified COG categories were metabolism, cellular processes and signaling, and information storage and processing. These findings correlate with several studies where different full-scale biogas reactors (fed with agricultural material, manure, wastewater sludge or municipal sludge) were functionally analyzed. Metabolic functions with 43 to 45% of the total reads, cellular processes and signaling (about 20% of the total reads), and basic house-keeping genes, e.g., information storage and processing (about 22% of the total reads) were the major COG categories (Guo et al. 2015; Cai et al. 2016). Within the COG category related to metabolism, the most abundant sub-categories were energy production and conversion, amino acid transport and metabolism, carbohydrate transport and metabolism, as well as lipid transport and metabolism. These metabolic functions are linked to the conversion of the fed substrates into smaller molecules and finally to methane (Schlüter et al. 2008; Li et al. 2013; Guo et al. 2015; Cai et al. 2016; Maus et al. 2016b). Among the amino acid transport and metabolism COG sub-category, genes involved in the biosynthesis of valine, leucine, and isoleucine as well as the metabolism of glycine, serine, threonine, cysteine, and methionine were identified (Guo et al. 2015). These amino acids are known to be commonly involved in Stickland reactions, indicating importance of amino acid fermentation pathways in the analyzed biogas reactors (Guo et al. 2015). Reads assigned to the carbohydrate metabolism COG category referred to genes of the sub-categories glycolysis/gluconeogenesis, pentose phosphate pathway, and amino sugar and nucleotide sugar metabolism (Guo et al. 2015) as well as processing of monosaccharides and disaccharides (Schlüter et al. 2008). These findings indicated that abundant species within the analyzed biogas reactors are involved in carbohydrate digestion and energy conversion. Additionally, assignments to enzymes like cellobiose phosphorylase, glucosidase, and cellulase/cellobiase indicated the potential for cellulose degradation within agricultural BGPs (Jaenicke et al. 2011; Maus et al. 2016b).

Several studies analyzing different full-scale anaerobic digesters (fed with manure or sludge from wastewater treatment plants and operated under mesophilic or thermophilic conditions) revealed highly similar results for the major level 1 SEED sub-systems. These refer to metabolism of carbohydrates, followed by clustering-based systems, protein metabolism, and amino acids and derivatives (Yang et al. 2014b; Guo et al. 2015; Luo et al. 2016). These major subsystems were also identified in other microbiomes from different ecosystems, like soil, freshwater or wastewater treatment plants and most of them can be related to the degradation of organic matter which occurs in many different habitats (Cai et al. 2016). However, differences in the functional genes were uncovered for anaerobic digesters fed with manure or activated wastewater sludge. The metagenomes of the sludge-based digesters featured significantly higher abundances of genes involved in nitrogen metabolism, phosphorus metabolism, and aromatic compound metabolism. These findings correlate with the composition of the wastewater sludge, where higher amounts of nitrite, aromatic compounds, organic contaminants, and phosphate are present compared to manure (Luo et al. 2016).

Since the carbohydrate metabolism was shown to be the major SEED level 1 subsystem for several different anaerobic digesters, detailed analyses of this sub-system on level 2 were conducted. The major level 2 carbohydrate sub-systems refer to the central carbohydrate metabolism and one-carbon metabolism (Yang et al. 2014b; Guo et al. 2015). Identified functions comprise transport and oxidation of main carbon sources, which are then enzymatically converted into metabolic precursors for the generation of cell biomass (Yang et al. 2014b). Especially the one-carbon metabolism plays an important role in the methanogenesis process (Ferry 1999).

Assignment of metagenome reads to KEGG metabolic pathways or Pfam families led to the identification of the major methanogenesis pathway performed in the analyzed biogas reactors (Yang et al. 2014b; Guo et al. 2015; Cai et al. 2016; Luo et al. 2016; Stolze et al. 2015). In principle, three methane synthesis pathways were described, namely hydrogenotrophic, acetoclastic, and methylotrophic methanogenesis. For biogas reactors fed with, e.g., sewage sludge, it was shown that the abundance of genes involved in acetoclastic methanogenesis was higher than the abundance of genes involved in hydrogenotrophic methane synthesis (Yang et al. 2014b; Guo et al. 2015; Cai et al. 2016). In contrast, genes involved in the hydrogenotrophic pathway were more abundant in biogas reactors fed with agricultural residues, maize silage, and manure (Stolze et al. 2015; Jaenicke et al. 2011). The methylotrophic pathway seems to be of minor importance in the analyzed biogas reactors (Guo et al. 2015).

The most abundant genes within the acetoclastic pathway were acetyl-CoA synthetase (EC: 6.2.1.1) and acetyl-CoA decarbonylase/synthase complex (ACDS) (Yang et al. 2014b; Luo et al. 2016). These genes are essential in the synthesis of acetyl-CoA from acetate (Li et al. 2013). Since these genes are also present in *Bacteria* where they are involved in other metabolic pathways, the identification of the major methanogenesis pathway based on the abundance of these genes is biased (Luo et al. 2016). On the other hand, the most dominant genes of the hydrogenotrophic pathway were formate dehydrogenase (EC: 1.2.1.2) and formylmethanofuran dehydrogenase (EC: 1.2.99.5) (Yang et al. 2014b; Luo et al. 2016).

Functional interpretation of metagenomes representing biogas communities elucidated their functional potential, which strongly depends on the fed substrates, but does not provide insights into the metabolically active community which in principle can be tackled by metatranscriptome/metaproteome analyses (see below) (Yang et al. 2014b; Guo et al. 2015; Cai et al. 2016).

### Single-read assembly and contig binning to compile genomes of biogas community members

A pilot study on functional characterization of the biogas microbiome employing a novel metagenome assembly and binning strategy was carried out by Campanaro et al. (2016). These authors analyzed laboratory-scale continuously stirred tank reactors (CSTRs) fed with cattle manure under thermophilic conditions. Approximately, 51 Gb metagenomic sequence information obtained on the Illumina HiSeq system was assembled and binned based on contig tetranucleotide frequencies and coverage of contigs by sequence reads. Coverage information resulted from metagenome sequencing of eight reactors varying in their OLRs assuming that different OLRs affect abundances of taxa residing in the system. The binning approach resulted in the reconstruction of 106 MAGs taxonomically assigned to the phyla *Firmicutes*, *Proteobacteria*, *Bacteroidetes*, *Synergistetes*, *Actinobacteria*, *Thermotogae*, *Spirochaetes*, and *Euryarchaeota*. However, only ten of the 106 MAGs could be assigned at the genus level whereas most of them were only classified at higher taxonomic ranks indicating that currently, genomes closely related to the newly reconstructed biogas MAGs are missing in databases. This means that the analyzed biogas community is a rich source of new, so far uncharacterized species that probably cannot easily be obtained by cultivation-based approaches. The three most abundant species represented by MAGs in the system were assigned to the *Clostridiales* (phylum *Firmicutes*), *Methanoculleus* sp. (phylum *Euryarchaeota*), and *Rikenellaceae* (phylum *Bacteroidetes*).

In this regard, a new *Methanoculleus* species, tentatively designated Candidatus *Methanoculleus thermohydrogenotrophicum* (sp. nov.) was reconstructed. This new species reached dominance levels of up to 20% in a particular thermophilic biogas reactor (Kougias et al. 2017). The dominance of species belonging to the genus *Methanoculleus* has previously been reported for mesophilic as well as thermophilic biogas systems (Jaenicke et al. 2011; Stolze et al. 2015; Maus et al. 2016b; Maus et al. 2017) (also refer to chapter 2.2.). The assembly/binning approach also led to the identification of other new taxa of higher ranks. This was exemplified by recognition of one MAG assigned to the phylum *Euryarchaeota*. The corresponding, so far uncultured species most probably represents a new class of this phylum.

Moreover, the compilation of MAG sequence information allows for reconstruction of corresponding metabolic pathways based on the organism’s genome. Species represented by MAGs were classified according to key pathways of the biogas process such as carbohydrate utilization, fatty acid degradation, amino acid fermentation, propionate and butanoate metabolism, acetogenesis, and methanogenesis. As perspective, functional interactions between biogas community members can be deduced based on MAG derived sequence information.

A similar metagenomic assembly/binning approach was conducted for mesophilic and thermophilic two-stage lab-scale CSTRs digesting cattle manure (Treu et al. 2016b; Bassani et al. 2015). This study yielded 157 new MAGs. However, settings for compilation of MAGs were less stringent compared to the previous study (completeness higher than 20% and contamination rate lower than 50%). Overall, the taxonomic distribution of identified taxa was similar to the study described above. However, also rare biogas taxa representing the phyla *Acidobacteria*, *Fibrobacteres*, *Lentisphaerae*, *Planctomycetes*, and *Thermotogae* were identified in the set of MAGs. Moreover, comparative analyses enabled to determine a core group of microorganisms that are important for the biogas process comprising among others *Methanoculleus*, *Methanothermobacter*, *Synthrophomonas*, and *Proteobacteria*.

First metagenome assembly for a full-scale mesophilic BGP fed with maize silage and pig manure was published in 2015 featuring a sequencing depth of 17 Gb (Bremges et al. 2015). This approach yielded an assembly size of 228 Mb; and 250,596 protein-coding genes were predicted on assembled continuous sequences. As an example, almost all gene products of the KEGG (Kyoto Encyclopedia of Genes and Genomes) pathway map “Methane Metabolism” are encoded on the compiled contigs. Reuse of these data was encouraged since all analysis tools and nucleotide sequences were provided as one Docker container accessible at “Docker-Hub” (Docker-Hub Registry).

Likewise, the microbiome of a mesophilic (40 °C) agricultural full-scale BGP loaded with maize silage and farm animal manure was analyzed by metagenome sequencing (Güllert et al. 2016). Metagenomic sequence reads were assembled to a size of 1.25 Gb comprising approx. two million predicted coding regions. Binning of contigs based on sequence composition and differential coverage resulted in 104 high-quality MAGs representing the taxa *Firmicutes*, *Bacteroidetes*, *Fibrobacteres*, *Spirochaetes*, *Actinobacteria*, *Verrucomicrobia*, and *Euryarchaeota*. Only very few MAGs could be assigned at the species or genus level. Examples are MAGs classified to belong to the genera *Fibrobacter*, *Clostridium*, *Paludibacter*, *Methanosarcina*, and the family *Porphyromonadaceae*. MAGs assigned to the genus *Clostridium* and the family *Lachnospiraceae* (phylum *Firmicutes*) possess scaffoldin genes encoding the structural scaffoldin subunit of cellulosomes. This finding indicates that the corresponding, so far unknown *Bacteria* are able to hydrolyze cellulose by employment of cellulosomes. Availability of the identified MAGs now enables analysis of the genomic context regarding the utilization potential of complex carbohydrates.

The to date deepest metagenome sequencing was performed for three mesophilic and one thermophilic full-scale BGPs digesting renewable raw materials, mainly maize silage, together with different types of manure (Stolze et al. 2016). In total, 328 Gb sequence information was obtained for these four BGPs yielding an assembly size of approx. 1.5 Gb. Assembled contigs could be bundled to 532 MAGs, five of which represented noticeable distinct taxa of the BGPs analyzed. The latter five MAGs were assigned to the phyla *Thermotogae*, *Fusobacteria*, *Spirochaetes*, and *Cloacimonetes*. Reliability of the assembly/binning approach could be demonstrated by the example of the *Thermotogae* MAG since it is very closely related to the isolate *Defluviitoga tunisiensis* L3 obtained from the same thermophilic BGP sampled for metagenome sequencing (Maus et al. 2016b). The other reconstructed MAGs represent novel and uncharacterized species. Based on the MAG sequence information, the metabolism of the corresponding species was reconstructed allowing classification of the *Fusobacteria* and *Cloacimonetes* taxonomic units as amino acid fermenting and carbon dioxide/hydrogen producing *Bacteria*, whereas the *Thermotogae* and *Spirochaetes* MAGs were predicted to represent sugar utilizing and acetate, carbon dioxide, and hydrogen producing *Bacteria*. Moreover, obtained results indicated a syntrophic association for the taxa analyzed. The described studies impressively illustrate the usefulness of metagenome assemblies combined with binning methods to uncover so-far unknown species of the biogas microbiome residing in biogas reactor systems.

The metagenome assembly/binning approach has also been applied for other anaerobic digestion habitats to reconstruct MAGs representing novel taxa. For example, three MAGs assigned to the new candidate phylum Hyd24-12 of the superphylum *Fibrobacteres-Chlorobi-Bacteroidetes* were reconstructed from metagenome sequences originating from full-scale mesophilic digesters of wastewater treatment plants (Kirkegaard et al. 2016). Hyd24-12 members were predicted to produce acetate and hydrogen by fermenting sugars and may utilize sulfur as terminal electron acceptor. Likewise, five MAGs representing members of the insufficiently analyzed class *Anaerolinea* (phylum *Chloroflexi*) were obtained from lab-scale thermophilic cellulose-fermenting reactors (Xia et al. 2014; Xia et al. 2016). Reconstruction of their metabolism indicates a carbohydrate-based lifestyle with acetate, lactate, and hydrogen as fermentation end-products.

In summary, metagenome assembly and binning approaches contributed and still will contribute to the compilation of a resource covering genome sequence information of biogas community members that so far have not been cultivated. Future metagenome assembly/binning approaches will benefit from sequencing technologies producing longer read lengths such as single molecule real-time (SMRT) sequencing (PacBio©). Longer reads significantly enhance taxonomic binning and genome compilation as recently exemplified for a microbiome sample from a commercial biogas reactor fed with slaughterhouse waste, food waste, and plant biomass located in Sweden (Frank et al. 2016).

## The functional characterization of microbial communities residing in biogas plants by analyzing community RNA and community proteins

**Fig. 5:**
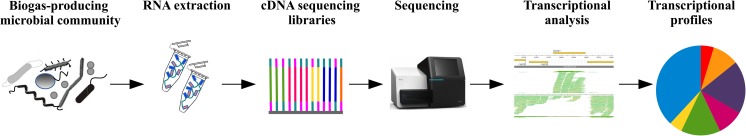
Metatranscriptome-based analyses of biogas-producing microbial communities. After sampling, whole community RNA was extracted followed by depletion of ribosomal RNAs. Metatranscriptome cDNA libraries were prepared and sequenced. Resulting metatranscriptome reads were mapped on corresponding metagenome data or MAGs. Finally, Transcripts per million (TPM) values were calculated for each gene to deduce transcriptional profiles of biogas microorganisms

**Fig. 6:**
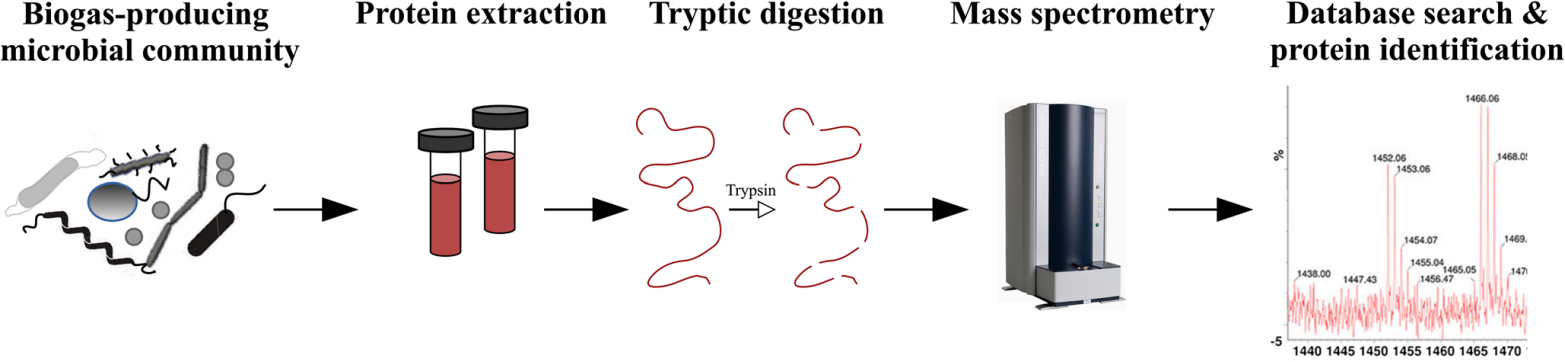
Metaproteomics workflow comprising sampling of the microbial biogas community, protein extraction, tryptic digestion of proteins, mass spectrometry of resulting peptides, and database searching using mass spectrometry data to identify proteins within the metaproteome analyzed

Metagenome analyses for biogas communities revealed their genetic potential. However, they do not enable conclusions on the metabolic activity of community members. To tackle profiling of the metabolically active biogas community, metatranscriptome, and metaproteome analyses were conducted. Metatranscriptome analyses provided insights into the transcriptional activity of biogas microbiomes (see Fig. 5). However, expression of enzymes, the catalysts of metabolism, involves translation of messenger-RNAs implicating the possibility of regulation at the post-transcriptional level. Analysis of the biogas microbiome’s proteome was addressed in metaproteome studies (see Fig. 6).

### The functional characterization of biogas communities by sequencing the metatranscriptome

First high-throughput metatranscriptome sequencing for a full-scale BGP fed with renewable primary products and manure was done based on total RNA preparations from biogas community members (Zakrzewski et al. 2012). Since the majority of transcriptome reads represented ribosomal RNAs, 16S rRNA tags were used for profiling of the transcriptionally active community members. Most sequences were assigned to the phyla *Euryarchaeota* and *Firmicutes* followed by *Bacteroidetes*, *Actinobacteria*, and *Synergistetes* indicating metabolic activity of microorganisms belonging to these phyla. Among the messenger-RNA (mRNA) tags within the latter dataset, sequences assigned to genes with predicted functions in hydrolysis, volatile fatty acid (VFA) and acetate formation, and methanogenesis were identified. High relative abundance of transcripts encoding key methanogenesis enzymes such as the methyl-coenzyme-M-reductase indicated high activity of hydrogenotrophic archaeal species in the analyzed BGP.

These results were confirmed in an independent study for another agricultural BGP fed with maize silage, cow and chicken manure (Güllert et al. 2016). In addition, *Bacteria* of the family *Peptococcaceae* and the order *Halanaerobiales* appeared to be transcriptionally very active in the latter study. Members of the *Firmicutes* residing in the biogas fermenter actively transcribed a diverse set of genes encoding glycosyl hydrolases some of which are involved in hydrolysis of lignocellulose. The authors of the latter study also took advantage of MAGs compiled from corresponding metagenome sequence data. In a genome-enabled metatranscriptomics approach, they mapped transcriptome sequences to MAGs allowing detection of putatively cellulolytic Polysaccharide Utilization Loci (PUL) in bacteroidetal MAGs. Likewise, genes for cellulosome-associated cellulases were predominantly transcribed in MAGs assigned to the *Firmicutes* (Güllert et al. 2016). Importance of PUL identified in *Bacteroidetes* members for polysaccharide decomposition was also shown for a thermophilic lab-scale reactor with microcrystalline cellulose as substrate by analyzing the microbiome’s metatranscriptome (Xia et al. 2014).

Metatranscriptome analyses were also reported for a thermophilic full-scale BGP digesting maize silage, barley, and cattle manure (Maus et al. 2016b). Whole metatranscriptome sequencing without prior removal of ribosomal RNAs revealed high transcriptional activities of the taxa *Defluviitoga* (*Thermotogae*), *Methanoculleus* (*Euryarchaeota*), *Clostridium* cluster III (*Firmicutes*), *Tepidanaerobacter* (*Firmicutes*), *Anaerobaculum* (*Synergistetes*), and *Cellulosibacter* (*Firmicutes*) in decreasing order regarding their contribution to 16S rRNA-derived sequence tags. In contrast, members of the genus *Halocella* and other genera appeared to be transcriptionally less active compared to their relative abundance estimated by evaluation of corresponding metagenome-derived 16S rRNA gene sequences. Importance of *Defluviitoga* species for the thermophilic full-scale biogas process with renewable primary products as substrates was previously shown (Maus et al. 2016b).

The genome-enabled metatranscriptomics approach also was exemplified in a very recent study in which metatranscriptome data from full-scale BGPs were mapped to metagenomically assembled and binned genomes representing distinctively abundant taxa (Stolze et al. 2016). Evaluation of transcriptional activities revealed that the metabolism of two MAGs assigned to the phyla *Thermotogae* and *Spirochaetes* is based on sugar utilization whereas amino acid fermentation was predicted for a *Fusobacteria* and a *Cloacimonetes* MAG.

Further metatranscriptome studies were conducted for lab-scale biogas systems addressing specific questions. Treu et al. (2016a) analyzed the effect of long-chain-fatty-acid (LCFA) addition to the biogas microbiome at the transcriptional level. Genes of species belonging to the genus *Syntrophomonas* were upregulated in response to the feeding of LCFA indicating that these species are important for LCFA degradation. The underlying analysis also took advantage of the availability of *Synthrophomonas* sp. MAGs compiled in an accompanying study (Campanaro et al. 2016). Genome-enabled metatranscriptomics elucidated the transcriptional profile of *Synthrophomonas* sp. under the conditions tested. Other community members expressed protective mechanisms towards the effects of LCFA. Likewise, the influence of the process temperature was investigated at the example of lab-scale biogas reactors fermenting swine manure at 25 to 55 °C (Lin et al. 2016). Metatranscriptome sequencing revealed that methane production was regulated by limiting the diversity of functional pathways at higher temperatures. This means that functional pathways are centralized under thermophilic conditions. This observation correlates with lower diversities of thermophilic biogas microbiomes (see above).

### The functional characterization of biogas communities by analyzing the metaproteome

First metaproteome analyses were conducted for a thermophilic lab-scale biogas reactor fed with beet silage and rye (Hanreich et al. 2012). Separation of total protein preparations by 2-dimensional gel electrophoresis and subsequent analysis of tryptically digested peptides by nanoHPLC-nanoESI-MS/MS led to the identification of housekeeping proteins and enzymes assigned to the archaeal acetoclastic and hydrogenotrophic methanogenesis pathways demonstrating high activity of these routes under thermophilic conditions.

Likewise, metaproteome analyses were done for a lab-scale biogas system digesting the lignocellulose-rich substrates straw and hay to study hydrolysis pathways (Hanreich et al. 2013). *Bacteroidetes* members were found to express ABC transporter proteins predicted to be involved in sugar uptake and TonB-dependent receptors which probably represent components encoded by PUL. *Firmicutes* taxa appeared to be responsible for cellulose degradation whereas species of the *Bacteroidetes* mainly participated in the digestion of other polysaccharides. Again, key methanogenesis enzymes were found to be highly expressed by species of the genera *Methanobacterium*, *Methanosaeta*, and *Methanoculleus*.

Higher throughput regarding identification of non-redundant protein functions within the metaproteome of biogas-producing microbial communities was achieved for a laboratory biogas system digesting office paper and utilizing the organic fraction of municipal solid waste as inoculum. Detected proteins covered *i.a.* the central carbon metabolism, fermentation, acetogenesis, syntrophic acetate oxidation, proteolysis, and methanogenesis (Lü et al. 2014).

Subsequently, first metaproteome analyses were performed for full-scale agricultural BGPs (Heyer et al. 2013). Obtained protein profiles allowed differentiation of mesophilic and thermophilic processes. Moreover, progression of the biogas process could be followed enabling for example prediction of disturbances such as acidification. Abundance of the key methanogenesis enzyme methyl-coenzyme-M-reductase assigned to the order *Methanosarcinales* decreased prior to acidification of the reactor and a decline of the methane yield (Heyer et al. 2013).

Improvements of metaproteome analyses of biogas communities were achieved by implementing different separation dimensions prior to protein identification. For example, application of liquid isoelectric focusing (IEF) as one of three dimensions resulted in the identification of approx. 750 to 1650 proteins within the metaproteome of communities residing in a mesophilic and a thermophilic BGP (Kohrs et al. 2014). Enzymes assigned to the four common phases of anaerobic digestion could be recognized in this approach and enabled differentiation of the mesophilic vs. the thermophilic process.

A more comprehensive study comprising 35 full-scale BGPs was conducted recently (Heyer et al. 2016). Processing of samples was done without elaborate pre-fractionation since high coverage of protein identifications could be achieved by applying the sensitive Orbitrap-mass-spectrometry technology. Proteotyping revealed different profile clusters for mesophilic and thermophilic BGPs and upflow anaerobic sludge blanket (UASB) fermenters and those fed with sewage sludge. Correlations between expressed proteins and the process parameters (i) ammonia concentration, (ii) sludge retention time, (iii) OLR, and (iv) temperature were detected. Very pronounced, high ammonia concentrations are associated with protein profiles indicating hydrogenotrophic methanogenesis and syntrophic acetate oxidation (Heyer et al. 2016).

Compilation of protein databases specific for biogas communities essentially improved protein identification in metaproteome analyses (Heyer et al. 2016; Ortseifen et al. 2016). Metagenome sequences representing biogas-producing microbial communities were assembled to deduce encoded gene products for compilation of biogas-specific protein databases. Metagenome assemblies also paved the way for obtaining genetic context information regarding proteins identified in metaproteome analyses. Metagenome contigs encoding metaproteome proteins provide information on genes located in the vicinity of the target gene. Exemplarily, an assembled contig from an agricultural BGP assigned to the genus *Methanoculleus* encoded several methanogenesis enzymes, three of which also were detected as abundant proteins in the community’s metaproteome (Ortseifen et al. 2016).

Quantitative metaproteomics applying nano-liquid-chromatography-(LC)-MS/MS elucidated the degradation of lignocellulosic biomass at the example of corn stover as substrate (Zhu et al. 2016). A great diversity of enzymes predicted to be involved in xylan degradation was identified. Most of these enzymes including xylanases, xylosidases, and cellulases were secreted by members of the *Firmicutes*. Interactions between bacterial species and enzymatic synergism with respect to hemicellulose digestion were elucidated in that study.

Likewise, in a bioprospecting approach involving induction of enzyme expression in response to cellulose addition, several enzymes predicted to be associated with cellulose metabolism were identified (Speda et al. 2017). For example, different cellulases, 1,4-β-cellobiosidases, endo 1-4-β-xylanases, cellobiose phosphorylases, and glycosyl hydrolase family proteins were identified in the extracellular metaproteome of the community. Since a corresponding metagenome had not been sequenced in that study, genes encoding enzymes of interest were not accessible, again demonstrating the advantages of combined metagenome/metaproteome analyses.

In a very recent study, the metaproteome of a community digesting grass in a two-stage biogas production system was analyzed under mesophilic vs. thermophilic conditions addressing the acidogenesis phase of the decomposition process (Abendroth et al. 2017). The metaproteome reflected the microbiome’s activity in polysaccharide utilization and sugar fermentation leading to the formation of short-chain fatty acids. Compared to mesophilic process conditions, the thermophilic community (55 °C) expressed a more stable protein profile suggesting that the thermophilic process early reached its steady state.

Commonly, metatranscriptome and/or metaproteome studies are complemented by metabolome analyses to correlate gene expression to metabolite profiles. However, to our knowledge, elaborate metabolome profiling mainly has been done for laboratory biogas-producing systems. A recent study revealed more than 200 metabolite peaks for a two stage lab-scale fermentation reactor digesting corn stalk (Yang et al. 2014a). The acidogenic phase was characterized by high levels of fatty acids whereas during methanogenesis, sugars, and sugar alcohols accumulated. The authors concluded that metabolome analyses should be interpreted considering metagenome data of the biogas community studied.

## Future prospects

Analyses of biogas producing communities by integrating findings from genome and metagenome sequencing, metatranscriptomics, metaproteomics, and metabolomics approaches provided deep insights into their compositions, performance, interrelations between community members, and dependencies concerning fed substrates and process parameters. Due to further improvements and developments in high-throughput sequencing technologies, large-scale taxonomic profiling will routinely be done for biogas communities to follow their succession in the course of fermentation processes and for monitoring purposes. In this context, full length HT-sequencing of the 16S rRNA marker gene on third- and fourth-generation sequencing platforms is of importance since longer sequences enable more precise taxonomic classifications.

Although hundreds of isolates were obtained from biogas producing communities by elaborate cultivation techniques, these do not represent their entire complexity. Cultivation of other community members may be challenging which most probably is due to specific growth requirements, trophic dependencies, and/or syntrophic associations. Moreover, it appeared that genome sequences of biogas producing microorganisms are underrepresented in public nucleotide sequence repositories implicating that more reference genomes are needed to evaluate metagenome, metatranscriptome, and metaproteome data from biogas microbiomes. Advanced bioinformatics strategies and deep metagenome sequencing led to assemblies and binning of genome sequences representing species that received little attention so far and/or are acting as key-players in AD. Accordingly, the latter approach provided access to the non-cultivable fraction of biogas communities.

Recently, a considerable number of MAGs from biogas communities was collected in databases and still offers the chance to discover new taxa. This resource, combined with metatranscriptome, metaproteome, and metabolome datasets, provides the opportunity to holistically study the metabolic potential and performance of taxa represented by MAGs and relate their activities to changing environmental conditions and process parameters. Increasingly, future research will also include network analyses since data collected by -omics technologies facilitate predictions concerning interactions between different taxa, trophic/syntrophic relationships, and other associations within the microbial community. Implementation of radio-isotopic labelling experiments even permits to follow the fate of particular metabolites and their fluxes.

In the future, metagenome assembly/binning studies will be complemented by single cell genomics (Yilmaz and Singh 2012) involving sorting and separation of single cells from complex communities followed by genome amplification (multiple displacement amplification - MDA), and NGS-sequencing. Single amplified genomes (SAGs) will complement the repository of biogas microbiome members. It can be expected that results from MAG and SAG analysis will also stimulate the development of new culturomics strategies to enable physiological analysis of new taxa (Lagier et al. 2015). Regarding exploitation and application of compiled knowledge on biogas communities, the design of *inocula* (starter) cultures for biogas processes as well as new and innovative approaches for monitoring, management, and engineering of anaerobic digestion assemblages is considered.

## Electronic supplementary material

ESM 1 (PDF 242 kb)

## References

[CR1] Abendroth C, Vilanova C, Günther T, Luschnig O, Porcar M (2015). Eubacteria and archaea communities in seven mesophile anaerobic digester plants in Germany. Biotechnol Biofuels.

[CR2] Abendroth C, Simeonov C, Peretó J, Antúnez O, Gavidia R, Luschnig O, Porcar M (2017). From grass to gas. Microbiome dynamics of grass biomass acidification under mesophilic and thermophilic temperatures. Biotechnol Biofuels.

[CR3] Akinosho H, Yee K, Close D, Ragauskas A (2014). The emergence of *Clostridium thermocellum* as a high utility candidate for consolidated bioprocessing applications. Front Chem.

[CR4] Akyol Ç, Aydin S, Ince O, Ince B (2016). A comprehensive microbial insight into single-stage and two-stage anaerobic digestion of oxytetracycline-medicated cattle manure. Chem Eng J.

[CR5] Angenent LT, Karim K, Al-Dahhan MH, Wrenn BA, Domíguez-Espinosa R (2004). Production of bioenergy and biochemicals from industrial and agricultural wastewater. Trends Biotechnol.

[CR6] Argyropoulos DN, Varzakas TH, John AH, Korres NE, O’Kiely P, Benzie JAH, West J (2013). Microbial communities and their dynamics in biomethane production. Bioenergy production by anearobic digestion. Using agricultural biomass and organic wastes.

[CR7] Attwood GT, Reilly K, Patel BK (1996). *Clostridium proteoclasticum* sp. nov., a novel proteolytic bacterium from the bovine rumen. Int J Syst Bacteriol.

[CR8] Bassani I, Kougias PG, Treu L, Angelidaki I (2015). Biogas upgrading via hydrogenotrophic methanogenesis in two-stage continuous stirred tank reactors at mesophilic and thermophilic conditions. Environ Sci Technol.

[CR9] Ben Hania W, Godbane R, Postec A, Hamdi M, Ollivier B, Fardeau M-L (2012). *Defluviitoga tunisiensis* gen. nov., sp. nov., a thermophilic bacterium isolated from a mesothermic and anaerobic whey digester. Int J Syst Evol Microbiol.

[CR10] de Bok FA, Stams AJ, Dijkema C, Boone DR (2001). Pathway of propionate oxidation by a syntrophic culture of *Smithella propionica* and *Methanospirillum hungatei*. Appl Environ Microbiol.

[CR11] Bremges A, Maus I, Belmann P, Eikmeyer F, Winkler A, Albersmeier A, Pühler A, Schlüter A, Sczyrba A (2015). Deeply sequenced metagenome and metatranscriptome of a biogas-producing microbial community from an agricultural production-scale biogas plant. Gigascience.

[CR12] Cabezas A, de Araujo JC, Callejas C, Galès A, Hamelin J, Marone A, Sousa DZ, Trably E, Etchebehere C (2015). How to use molecular biology tools for the study of the anaerobic digestion process?. Rev Environ Sci Biotechnol.

[CR13] Cai M, Wilkins D, Chen J, Ng S-K, Lu H, Jia Y, Lee PKH (2016). Metagenomic reconstruction of key anaerobic digestion pathways in municipal sludge and industrial wastewater biogas-producing systems. Front Microbiol.

[CR14] Campanaro S, Treu L, Kougias PG, de FD, Valle G, Angelidaki I (2016). Metagenomic analysis and functional characterization of the biogas microbiome using high throughput shotgun sequencing and a novel binning strategy. Biotechnol Biofuels.

[CR15] Caporaso JG, Kuczynski J, Stombaugh J, Bittinger K, Bushman FD, Costello EK, Fierer N, Peña AG, Goodrich JK, Gordon JI, Huttley GA, Kelley ST, Knights D, Koenig JE, Ley RE, Lozupone CA, McDonald D, Muegge BD, Pirrung M, Reeder J, Sevinsky JR, Turnbaugh PJ, Walters WA, Widmann J, Yatsunenko T, Zaneveld J, Knight R (2010). QIIME allows analysis of high-throughput community sequencing data. Nat Methods.

[CR16] Čater M, Fanedl L, Logar RM (2013). Microbial community analyses in biogas reactors by molecular methods. Acta Chim Slov.

[CR17] Carballa M, Regueiro L, Lema JM (2015). Microbial management of anaerobic digestion. Exploiting the microbiome-functionality nexus. Curr Opin Biotechnol.

[CR18] Cardinali-Rezende J, Colturato LFDB, Colturato TDB, Chartone-Souza E, Nascimento AMA, Sanz JL (2012). Prokaryotic diversity and dynamics in a full-scale municipal solid waste anaerobic reactor from start-up to steady-state conditions. Bioresour Technol.

[CR19] Caspi R, Billington R, Ferrer L, Foerster H, Fulcher CA, Keseler IM, Kothari A, Krummenacker M, Latendresse M, Mueller LA, Ong Q, Paley S, Subhraveti P, Weaver DS, Karp PD (2016). The MetaCyc database of metabolic pathways and enzymes and the BioCyc collection of pathway/genome databases. Nucleic Acids Res.

[CR20] Chang A, Scheer M, Grote A, Schomburg I, Schomburg D (2009). BRENDA, AMENDA and FRENDA the enzyme information system: new content and tools in 2009. Nucleic Acids Res.

[CR21] Cole JR, Tiedje JM (2014). History and impact of RDP: a legacy from Carl Woese to microbiology. RNA Biol.

[CR22] Collins G, Woods A, McHugh S, Carton MW, O’Flaherty V (2003). Microbial community structure and methanogenic activity during start-up of psychrophilic anaerobic digesters treating synthetic industrial wastewaters. FEMS Microbiol Ecol.

[CR23] Demirel B (2014). Major pathway of methane formation from energy crops in agricultural biogas digesters. Crit Rev Environ Sci Technol.

[CR24] Demirel B, Scherer P (2008). The roles of acetotrophic and hydrogenotrophic methanogens during anaerobic conversion of biomass to methane: a review. Rev Environ Sci Biotechnol.

[CR25] Deppenmeier U, Müller V, Gottschalk G (1996). Pathways of energy conservation in methanogenic archaea. Arch Mikrobiol.

[CR26] Dürre P (2005). Handbook on *clostridia*.

[CR27] Edgar RC (2010). Search and clustering orders of magnitude faster than BLAST. Bioinformatics.

[CR28] Edgar RC (2013). UPARSE. Highly accurate OTU sequences from microbial amplicon reads. Nat Methods.

[CR29] Ek A, Hallin S, Vallin L, Schnurer A, Karlsson M (2011) Slaughterhouse waste co-digestion—experiences from 15 years of full-scale operation. In: World Renewable Energy Congress - Sweden 8–13 May, 2011 : Volume 1 (Bioenergy Technology) Linköping University Electronic Press, Linköpings universitet, pp 64–71

[CR30] Fernandez AS, Hashsham SA, Dollhopf SL, Raskin L, Glagoleva O, Dazzo FB, Hickey RF, Criddle CS, Tiedje JM (2000). Flexible community structure correlates with stable community function in methanogenic bioreactor communities perturbed by glucose. Appl Environ Microbiol.

[CR31] Ferrari B, Winsley T, Ji M, Neilan B (2014). Insights into the distribution and abundance of the ubiquitous *candidatus Saccharibacteria* phylum following tag pyrosequencing. Sci Rep.

[CR32] Ferry JG (1999). Enzymology of one-carbon metabolism in methanogenic pathways. FEMS Microbiol Rev.

[CR33] Finn RD, Attwood TK, Babbitt PC, Bateman A, Bork P, Bridge AJ, Chang H-Y, Dosztányi Z, El-Gebali S, Fraser M, Gough J, Haft D, Holliday GL, Huang H, Huang X, Letunic I, Lopez R, Lu S, Marchler-Bauer A, Mi H, Mistry J, Natale DA, Necci M, Nuka G, Orengo CA, Park Y, Pesseat S, Piovesan D, Potter SC, Rawlings ND, Redaschi N, Richardson L, Rivoire C, Sangrador-Vegas A, Sigrist C, Sillitoe I, Smithers B, Squizzato S, Sutton G, Thanki N, Thomas PD, Tosatto SCE, Wu CH, Xenarios I, Yeh L-S, Young S-Y, Mitchell AL (2017). InterPro in 2017-beyond protein family and domain annotations. Nucleic Acids Res.

[CR34] FNR - Agency of renewable biomass of the German ministry for agriculture (2017) Bioenergy in Germany, facts and figures 2017. https://mediathek.fnr.de/bioenergy-in-germany-facts-and-figures.html. Accessed 22 March 2018

[CR35] Fonknechten N, Chaussonnerie S, Tricot S, Lajus A, Andreesen JR, Perchat N, Pelletier E, Gouyvenoux M, Barbe V, Salanoubat M, Le Paslier D, Weissenbach J, Cohen GN, Kreimeyer A (2010). *Clostridium sticklandii*, a specialist in amino acid degradation: revisiting its metabolism through its genome sequence. BMC Genomics.

[CR36] Fontana A, Patrone V, Puglisi E, Morelli L, Bassi D, Garuti M, Rossi L, Cappa F (2016). Effects of geographic area, feedstock, temperature, and operating time on microbial communities of six full-scale biogas plants. Bioresour Technol.

[CR37] Fotidis IA, Karakashev D, Angelidaki I (2014). The dominant acetate degradation pathway/methanogenic composition in full-scale anaerobic digesters operating under different ammonia levels. Int J Environ Sci Technol.

[CR38] Frank JA, Pan Y, Tooming-Klunderud A, Eijsink VGH, McHardy AC, Nederbragt AJ, Pope PB (2016). Improved metagenome assemblies and taxonomic binning using long-read circular consensus sequence data. Sci Rep.

[CR39] Franke-Whittle IH, Walter A, Ebner C, Insam H (2014). Investigation into the effect of high concentrations of volatile fatty acids in anaerobic digestion on methanogenic communities. Waste Manag.

[CR40] Goux X, Calusinska M, Lemaigre S, Marynowska M, Klocke M, Udelhoven T, Benizri E, Delfosse P (2015). Microbial community dynamics in replicate anaerobic digesters exposed sequentially to increasing organic loading rate, acidosis, and process recovery. Biotechnol Biofuels.

[CR41] Güllert S, Fischer MA, Turaev D, Noebauer B, Ilmberger N, Wemheuer B, Alawi M, Rattei T, Daniel R, Schmitz RA, Grundhoff A, Streit WR (2016). Deep metagenome and metatranscriptome analyses of microbial communities affiliated with an industrial biogas fermenter, a cow rumen, and elephant feces reveal major differences in carbohydrate hydrolysis strategies. Biotechnol Biofuels.

[CR42] Guo J, Peng Y, Ni B-J, Han X, Fan L, Yuan Z (2015). Dissecting microbial community structure and methane-producing pathways of a full-scale anaerobic reactor digesting activated sludge from wastewater treatment by metagenomic sequencing. Microb Cell Factories.

[CR43] Hahnke S, Striesow J, Elvert M, Mollar XP, Klocke M (2014). *Clostridium bornimense* sp. nov., isolated from a mesophilic, two-phase, laboratory-scale biogas reactor. Int J Syst Evol Microbiol.

[CR44] Hahnke S, Langer T, Koeck DE, Klocke M (2016). Description of *Proteiniphilum saccharofermentans* sp. nov., *Petrimonas mucosa* sp. nov. and *Fermentimonas caenicola* gen. nov., sp. nov., isolated from mesophilic laboratory-scale biogas reactors, and emended description of the genus Proteiniphilum. Int J Syst Evol Microbiol.

[CR45] Hahnke S, Langer T, Klocke M (2018). *Proteiniborus indolifex* sp. nov., isolated from a thermophilic industrial-scale biogas plant. Int J Syst Evol Microbiol.

[CR46] Han G, Shin SG, Lee J, Shin J, Hwang S (2017). A comparative study on the process efficiencies and microbial community structures of six full-scale wet and semi-dry anaerobic digesters treating food wastes. Bioresour Technol.

[CR47] Hanreich A, Heyer R, Benndorf D, Rapp E, Pioch M, Reichl U, Klocke M (2012). Metaproteome analysis to determine the metabolically active part of a thermophilic microbial community producing biogas from agricultural biomass. Can J Microbiol.

[CR48] Hanreich A, Schimpf U, Zakrzewski M, Schlüter A, Benndorf D, Heyer R, Rapp E, Pühler A, Reichl U, Klocke M (2013). Metagenome and metaproteome analyses of microbial communities in mesophilic biogas-producing anaerobic batch fermentations indicate concerted plant carbohydrate degradation. Syst Appl Microbiol.

[CR49] Harris JK, Kelley ST, Pace NR (2004). New perspective on uncultured bacterial phylogenetic division OP11. Appl Environ Microbiol.

[CR50] Heyer R, Kohrs F, Benndorf D, Rapp E, Kausmann R, Heiermann M, Klocke M, Reichl U (2013). Metaproteome analysis of the microbial communities in agricultural biogas plants. New Biotechnol.

[CR51] Heyer R, Benndorf D, Kohrs F, De Vrieze J, de Boon N, Hoffmann M, Rapp E, Schlüter A, Sczyrba A, Reichl U (2016). Proteotyping of biogas plant microbiomes separates biogas plants according to process temperature and reactor type. Biotechnol Biofuels.

[CR52] Huerta-Cepas J, Szklarczyk D, Forslund K, Cook H, Heller D, Walter MC, Rattei T, Mende DR, Sunagawa S, Kuhn M, Jensen LJ, von Mering C, Bork P (2016). eggNOG 4.5. A hierarchical orthology framework with improved functional annotations for eukaryotic, prokaryotic and viral sequences. Nucleic Acids Res.

[CR53] Huson DH, Beier S, Flade I, Górska A, El-Hadidi M, Mitra S, Ruscheweyh H-J, Tappu R (2016). MEGAN community edition—interactive exploration and analysis of large-scale microbiome sequencing data. PLoS Comput Biol.

[CR54] Jackson BE, Bhupathiraju VK, Tanner RS, Woese CR, McInerney MJ (1999). *Syntrophus aciditrophicus* sp. nov., a new anaerobic bacterium that degrades fatty acids and benzoate in syntrophic association with hydrogen-using microorganisms. Arch Mikrobiol.

[CR55] Jaenicke S, Ander C, Bekel T, Bisdorf R, Dröge M, Gartemann K-H, Jünemann S, Kaiser O, Krause L, Tille F, Zakrzewski M, Pühler A, Schlüter A, Goesmann A (2011). Comparative and joint analysis of two metagenomic datasets from a biogas fermenter obtained by 454-pyrosequencing. PLoS One.

[CR56] Jetten MSM, Stams AJM, Zehnder AJB (1990). Acetate threshold values and acetate activating enzymes in methanogenic bacteria. FEMS Microbiol Lett.

[CR57] Jünemann S, Kleinbölting N, Jaenicke S, Henke C, Hassa J, Nelkner J, Stolze Y, Albaum SP, Schlüter A, Goesmann A, Sczyrba A, Stoye J (2017). Bioinformatics for NGS-based metagenomics and the application to biogas research. J Biotechnol.

[CR58] Kanehisa M, Furumichi M, Tanabe M, Sato Y, Morishima K (2017). KEGG. New perspectives on genomes, pathways, diseases and drugs. Nucleic Acids Res.

[CR59] Karakashev D, Batstone DJ, Angelidaki I (2005). Influence of environmental conditions on methanogenic compositions in anaerobic biogas reactors. Appl Environ Microbiol.

[CR60] Kaspar HF, Wuhrmann K (1978). Kinetic parameters and relative turnovers of some important catabolic reactions in digesting sludge. Appl Environ Microbiol.

[CR61] Kern T, Linge M, Rother M (2015). *Methanobacterium aggregans* sp. nov., a hydrogenotrophic methanogenic archaeon isolated from an anaerobic digester. Int J Syst Evol Microbiol.

[CR62] Kern T, Fischer MA, Deppenmeier U, Schmitz RA, Rother M (2016). *Methanosarcina flavescens* sp. nov., a methanogenic archaeon isolated from a full-scale anaerobic digester. Int J Syst Evol Microbiol.

[CR63] Kim M, Oh H-S, Park S-C, Chun J (2014). Towards a taxonomic coherence between average nucleotide identity and 16S rRNA gene sequence similarity for species demarcation of prokaryotes. Int J Syst Evol Microbiol.

[CR64] Kirkegaard RH, Dueholm MS, McIlroy SJ, Nierychlo M, Karst SM, Albertsen M, Nielsen PH (2016). Genomic insights into members of the candidate phylum Hyd24-12 common in mesophilic anaerobic digesters. ISME J.

[CR65] Kirkegaard RH, McIlroy SJ, Kristensen JM, Nierychlo M, Karst SM, Dueholm MS, Albertsen M, Nielsen PH (2017). The impact of immigration on microbial community composition in full-scale anaerobic digesters. Sci Rep.

[CR66] Klocke M, Mähnert P, Mundt K, Souidi K, Linke B (2007). Microbial community analysis of a biogas-producing completely stirred tank reactor fed continuously with fodder beet silage as mono-substrate. Syst Appl Microbiol.

[CR67] Koch C, Müller S, Harms H, Harnisch F (2014). Microbiomes in bioenergy production. From analysis to management. Curr Opin Biotechnol.

[CR68] Koeck DE, Zverlov VV, Liebl W, Schwarz WH (2014). Comparative genotyping of *Clostridium thermocellum* strains isolated from biogas plants. Genetic markers and characterization of cellulolytic potential. Syst Appl Microbiol.

[CR69] Koeck DE, Ludwig W, Wanner G, Zverlov VV, Liebl W, Schwarz WH (2015). *Herbinix hemicellulosilytica* gen. nov., sp. nov., a thermophilic cellulose-degrading bacterium isolated from a thermophilic biogas reactor. Int J Syst Evol Microbiol.

[CR70] Koeck DE, Hahnke S, Zverlov VV (2016). *Herbinix luporum* sp. nov., a thermophilic cellulose-degrading bacterium isolated from a thermophilic biogas reactor. Int J Syst Evol Microbiol.

[CR71] Koeck DE, Mechelke M, Zverlov VV, Liebl W, Schwarz WH (2016). *Herbivorax saccincola* gen. nov., sp. nov., a cellulolytic, anaerobic, thermophilic bacterium isolated via in sacco enrichments from a lab-scale biogas reactor. Int J Syst Evol Microbiol.

[CR72] Kohrs F, Heyer R, Magnussen A, Benndorf D, Muth T, Behne A, Rapp E, Kausmann R, Heiermann M, Klocke M, Reichl U (2014). Sample prefractionation with liquid isoelectric focusing enables in depth microbial metaproteome analysis of mesophilic and thermophilic biogas plants. Anaerobe.

[CR73] Kougias PG, Campanaro S, Treu L, Zhu X, Angelidaki I (2017). A novel archaeal species belonging to *Methanoculleus* genus identified via de-novo assembly and metagenomic binning process in biogas reactors. Anaerobe.

[CR74] Krakat N, Westphal A, Satke K, Schmidt S, Scherer P (2010). The microcosm of a biogas fermenter. Comparison of moderate hyperthermophilic (60°C) with thermophilic (55°C) conditions. Eng Life Sci.

[CR75] Krause L, Diaz NN, Goesmann A, Kelley S, Nattkemper TW, Rohwer F, Edwards RA, Stoye J (2008). Phylogenetic classification of short environmental DNA fragments. Nucleic Acids Res.

[CR76] Lin Q, De Vrieze J, de He G, Li X, Li J (2016). Temperature regulates methane production through the function centralization of microbial community in anaerobic digestion. Bioresour Technol.

[CR77] Labbe RG, Duncan CL (1975). Influence of carbohydrates on growth and sporulation of *Clostridium perfringens* type A. Appl Microbiol.

[CR78] Lagier J-C, Hugon P, Khelaifia S, Fournier P-E, La Scola B, Raoult D (2015). The rebirth of culture in microbiology through the example of culturomics to study human gut microbiota. Clin Microbiol Rev.

[CR79] Lebuhn M, Hanreich A, Klocke M, Schlüter A, Bauer C, Pérez CM (2014). Towards molecular biomarkers for biogas production from lignocellulose-rich substrates. Anaerobe.

[CR80] Lee MJ, Zinder SH (1988). Hydrogen partial pressures in a thermophilic acetate-oxidizing methanogenic coculture. Appl Environ Microbiol.

[CR81] Lee J, Hwang B, Koo T, Shin SG, Kim W, Hwang S (2014). Temporal variation in methanogen communities of four different full-scale anaerobic digesters treating food waste-recycling wastewater. Bioresour Technol.

[CR82] Li A, Y’n C, Wang X, Ren L, Yu J, Liu X, Yan J, Zhang L, Wu S, Li S (2013). A pyrosequencing-based metagenomic study of methane-producing microbial community in solid-state biogas reactor. Biotechnol Biofuels.

[CR83] Lien T, Madsen M, Rainey FA, Birkeland NK (1998). *Petrotoga mobilis* sp. nov., from a North Sea oil-production well. Int J Syst Bacteriol.

[CR84] Liu WT, Marsh TL, Cheng H, Forney LJ (1997). Characterization of microbial diversity by determining terminal restriction fragment length polymorphisms of genes encoding 16S rRNA. Appl Environ Microbiol.

[CR85] Liu Z, DeSantis TZ, Andersen GL, Knight R (2008). Accurate taxonomy assignments from 16S rRNA sequences produced by highly parallel pyrosequencers. Nucleic Acids Res.

[CR86] Liu T, Sun L, Müller B, Schnürer A (2017). Importance of inoculum source and initial community structure for biogas production from agricultural substrates. Bioresour Technol.

[CR87] Lü F, Bize A, Guillot A, Monnet V, Madigou C, Chapleur O, Mazéas L, He P, Bouchez T (2014). Metaproteomics of cellulose methanisation under thermophilic conditions reveals a surprisingly high proteolytic activity. ISME J.

[CR88] Lucas R, Kuchenbuch A, Fetzer I, Harms H, Kleinsteuber S (2015) Long-term monitoring reveals stable and remarkably similar microbial communities in parallel full-scale biogas reactors digesting energy crops. FEMS Microbiol Ecol 91(3) 10.1093/femsec/fiv004 25764564

[CR89] Luo G, Fotidis IA, Angelidaki I (2016). Comparative analysis of taxonomic, functional, and metabolic patterns of microbiomes from 14 full-scale biogas reactors by metagenomic sequencing and radioisotopic analysis. Biotechnol Biofuels.

[CR90] Magoč T, Salzberg SL (2011). FLASH. Fast length adjustment of short reads to improve genome assemblies. Bioinformatics.

[CR91] Maus I, Cibis KG, Bremges A, Stolze Y, Wibberg D, Tomazetto G, Blom J, Sczyrba A, König H, Pühler A, Schlüter A (2016). Genomic characterization of *Defluviitoga tunisiensis* L3, a key hydrolytic bacterium in a thermophilic biogas plant and its abundance as determined by metagenome fragment recruitment. J Biotechnol.

[CR92] Maus I, Koeck DE, Cibis KG, Hahnke S, Kim YS, Langer T, Kreubel J, Erhard M, Bremges A, Off S, Stolze Y, Jaenicke S, Goesmann A, Sczyrba A, Scherer P, König H, Schwarz WH, Zverlov VV, Liebl W, Pühler A, Schlüter A, Klocke M (2016). Unraveling the microbiome of a thermophilic biogas plant by metagenome and metatranscriptome analysis complemented by characterization of bacterial and archaeal isolates. Biotechnol Biofuels.

[CR93] Maus I, Kim YS, Wibberg D, Stolze Y, Off S, Antonczyk S, Pühler A, Scherer P, Schlüter A (2017). Biphasic study to characterize agricultural biogas plants by high-throughput 16S rRNA gene amplicon sequencing and microscopic analysis. J Microbiol Biotechnol.

[CR94] McBee RH (1950). The anaerobic thermophilic cellulolytic bacteria. Bacteriol Rev.

[CR95] McBee RH (1954). The characteristics of *Clostridium thermocellum*. J Bacteriol.

[CR96] Meyer F, Paarmann D, D’Souza M, Olson R, Glass EM, Kubal M, Paczian T, Rodriguez A, Stevens R, Wilke A, Wilkening J, Edwards RA (2008). The metagenomics RAST server - a public resource for the automatic phylogenetic and functional analysis of metagenomes. BMC Bioinformatics.

[CR97] Moset V, Poulsen M, Wahid R, Højberg O, Møller HB (2015). Mesophilic versus thermophilic anaerobic digestion of cattle manure. Methane productivity and microbial ecology. Microb Biotechnol.

[CR98] Moyer CL, Dobbs FC, Karl DM (1994). Estimation of diversity and community structure through restriction fragment length polymorphism distribution analysis of bacterial 16S rRNA genes from a microbial mat at an active, hydrothermal vent system, Loihi Seamount, Hawaii. Appl Environ Microbiol.

[CR99] Müller B, Sun L, Westerholm M, Schnürer A (2016). Bacterial community composition and fhs profiles of low- and high-ammonia biogas digesters reveal novel syntrophic acetate-oxidising bacteria. Biotechnol Biofuels.

[CR100] Muyzer G, Waal EC, de Uitterlinden AG (1993). Profiling of complex microbial populations by denaturing gradient gel electrophoresis analysis of polymerase chain reaction-amplified genes coding for 16S rRNA. Appl Environ Microbiol.

[CR101] Nasir IM, Mohd Ghazi TI, Omar R (2012). Anaerobic digestion technology in livestock manure treatment for biogas production: a review. Eng Life Sci.

[CR102] Nettmann E, Bergmann I, Mundt K, Linke B, Klocke M (2008). Archaea diversity within a commercial biogas plant utilizing herbal biomass determined by 16S rDNA and mcrA analysis. J Appl Microbiol.

[CR103] Nettmann E, Bergmann I, Pramschüfer S, Mundt K, Plogsties V, Herrmann C, Klocke M (2010). Polyphasic analyses of methanogenic archaeal communities in agricultural biogas plants. Appl Environ Microbiol.

[CR104] Overbeek R, Olson R, Pusch GD, Olsen GJ, Davis JJ, Disz T, Edwards RA, Gerdes S, Parrello B, Shukla M, Vonstein V, Wattam AR, Xia F, Stevens R (2014). The SEED and the rapid annotation of microbial genomes using subsystems technology (RAST). Nucleic Acids Res.

[CR105] Ortseifen V, Stolze Y, Maus I, Sczyrba A, Bremges A, Albaum SP, Jaenicke S, Fracowiak J, Pühler A, Schlüter A (2016). An integrated metagenome and -proteome analysis of the microbial community residing in a biogas production plant. J Biotechnol.

[CR106] Ozbayram EG, Kleinsteuber S, Nikolausz M, Ince B, Ince O (2017). Effect of bioaugmentation by cellulolytic bacteria enriched from sheep rumen on methane production from wheat straw. Anaerobe.

[CR107] Peura S, Eiler A, Bertilsson S, Nykänen H, Tiirola M, Jones RI (2012). Distinct and diverse anaerobic bacterial communities in boreal lakes dominated by candidate division OD1. ISME J.

[CR108] Qiu Y-L, Hanada S, Ohashi A, Harada H, Kamagata Y, Sekiguchi Y (2008). *Syntrophorhabdus aromaticivorans* gen. Nov., sp. nov., the first cultured anaerobe capable of degrading phenol to acetate in obligate syntrophic associations with a hydrogenotrophic methanogen. Appl Environ Microbiol.

[CR109] Quast C, Pruesse E, Yilmaz P, Gerken J, Schweer T, Yarza P, Peplies J, Glöckner FO (2013). The SILVA ribosomal RNA gene database project. Improved data processing and web-based tools. Nucleic Acids Res.

[CR110] Ranjan R, Rani A, Metwally A, McGee HS, Perkins DL (2016). Analysis of the microbiome. Advantages of whole genome shotgun versus 16S amplicon sequencing. Biochem Biophys Res Commun.

[CR111] Rui J, Li J, Zhang S, Yan X, Wang Y, Li X (2015). The core populations and co-occurrence patterns of prokaryotic communities in household biogas digesters. Biotechnol Biofuels.

[CR112] Schloss PD, Westcott SL, Ryabin T, Hall JR, Hartmann M, Hollister EB, Lesniewski RA, Oakley BB, Parks DH, Robinson CJ, Sahl JW, Stres B, Thallinger GG, van Horn DJ, Weber CF (2009). Introducing mothur. Open-source, platform-independent, community-supported software for describing and comparing microbial communities. Appl Environ Microbiol.

[CR113] Schlüter A, Bekel T, Diaz NN, Dondrup M, Eichenlaub R, Gartemann K-H, Krahn I, Krause L, Krömeke H, Kruse O, Mussgnug JH, Neuweger H, Niehaus K, Pühler A, Runte KJ, Szczepanowski R, Tauch A, Tilker A, Viehöver P, Goesmann A (2008). The metagenome of a biogas-producing microbial community of a production-scale biogas plant fermenter analysed by the 454-pyrosequencing technology. J Biotechnol.

[CR114] Schnellen CGTP (1947) Onderzoekingen over de methaangisting. PhD thesis, Technical University, Delft

[CR115] Schnürer A (2016). Biogas production. Microbiology and technology. Adv Biochem Eng Biotechnol.

[CR116] Schnürer A, Nordberg A (2008). Ammonia, a selective agent for methane production by syntrophic acetate oxidation at mesophilic temperature. Water Sci Technol.

[CR117] Schnürer A, Zellner G, Svensson BH (1999). Mesophilic syntrophic acetate oxidation during methane formation in biogas reactors. FEMS Microbiol Ecol.

[CR118] Silvey P, Pullammanappallil PC, Blackall L, Nichols P (2000). Microbial ecology of the leach bed anaerobic digestion of unsorted municipal solid waste. Water Sci Technol.

[CR119] Simankova MV, Chernych NA, Osipov GA, Zavarzin GA (1993). *Halocella cellulolytica* gen. nov., sp. nov., a new obligately anaerobic, halophilic, cellulolytic bacterium. Syst Appl Microbiol.

[CR120] Simó C, Cifuentes A, García-Cañas V (2014). Fundamentals of advanced omics technologies.

[CR121] Söhngen C, Podstawka A, Bunk B, Gleim D, Vetcininova A, Reimer LC, Ebeling C, Pendarovski C, Overmann J (2016). BacDive—the bacterial diversity metadatabase in 2016. Nucleic Acids Res.

[CR122] Speda J, Jonsson B-H, Carlsson U, Karlsson M (2017). Metaproteomics-guided selection of targeted enzymes for bioprospecting of mixed microbial communities. Biotechnol Biofuels.

[CR123] Stolze Y, Zakrzewski M, Maus I, Eikmeyer F, Jaenicke S, Rottmann N, Siebner C, Pühler A, Schlüter A (2015). Comparative metagenomics of biogas-producing microbial communities from production-scale biogas plants operating under wet or dry fermentation conditions. Biotechnol Biofuels.

[CR124] Stolze Y, Bremges A, Rumming M, Henke C, Maus I, Pühler A, Sczyrba A, Schlüter A (2016). Identification and genome reconstruction of abundant distinct taxa in microbiomes from one thermophilic and three mesophilic production-scale biogas plants. Biotechnol Biofuels.

[CR125] St-Pierre B, Wright A-DG (2013). Metagenomic analysis of methanogen populations in three full-scale mesophilic anaerobic manure digesters operated on dairy farms in Vermont, USA. Bioresour Technol.

[CR126] St-Pierre B, Wright A-DG (2014). Comparative metagenomic analysis of bacterial populations in three full-scale mesophilic anaerobic manure digesters. Appl Microbiol Biotechnol.

[CR127] Sun W, Yu G, Louie T, Liu T, Zhu C, Xue G, Gao P (2015). From mesophilic to thermophilic digestion. The transitions of anaerobic bacterial, archaeal, and fungal community structures in sludge and manure samples. Appl Microbiol Biotechnol.

[CR128] Sun L, Liu T, Müller B, Schnürer A (2016). The microbial community structure in industrial biogas plants influences the degradation rate of straw and cellulose in batch tests. Biotechnol Biofuels.

[CR129] Sundberg C, Al-Soud WA, Larsson M, Alm E, Yekta SS, Svensson BH, Sørensen SJ, Karlsson A (2013). 454 pyrosequencing analyses of bacterial and archaeal richness in 21 full-scale biogas digesters. FEMS Microbiol Ecol.

[CR130] Takahashi S, Tomita J, Nishioka K, Hisada T, Nishijima M (2014). Development of a prokaryotic universal primer for simultaneous analysis of bacteria and archaea using next-generation sequencing. PLoS One.

[CR131] Tatusov RL, Galperin MY, Natale DA, Koonin EV (2000). The COG database. A tool for genome-scale analysis of protein functions and evolution. Nucleic Acids Res.

[CR132] Theuerl S, Kohrs F, Benndorf D, Maus I, Wibberg D, Schlüter A, Kausmann R, Heiermann M, Rapp E, Reichl U, Pühler A, Klocke M (2015). Community shifts in a well-operating agricultural biogas plant. How process variations are handled by the microbiome. Appl Microbiol Biotechnol.

[CR133] Tremblay J, Singh K, Fern A, Kirton ES, He S, Woyke T, Lee J, Chen F, Dangl JL, Tringe SG (2015). Primer and platform effects on 16S rRNA tag sequencing. Front Microbiol.

[CR134] Treu L, Campanaro S, Kougias PG, Zhu X, Angelidaki I (2016). Untangling the effect of fatty acid addition at species level revealed different transcriptional responses of the biogas microbial community members. Environ Sci Technol.

[CR135] Treu L, Kougias PG, Campanaro S, Bassani I, Angelidaki I (2016). Deeper insight into the structure of the anaerobic digestion microbial community; the biogas microbiome database is expanded with 157 new genomes. Bioresour Technol.

[CR136] Vanwonterghem I, Jensen PD, Dennis PG, Hugenholtz P, Rabaey K, Tyson GW (2014). Deterministic processes guide long-term synchronised population dynamics in replicate anaerobic digesters. ISME J.

[CR137] Venkiteshwaran K, Bocher B, Maki J, Zitomer D (2015). Relating anaerobic digestion microbial community and process function. Microbiol Insights.

[CR138] Větrovský T, Baldrian P (2013). The variability of the 16S rRNA gene in bacterial genomes and its consequences for bacterial community analyses. PLoS One.

[CR139] De Vrieze J, de Hennebel T, Boon N, Verstraete W (2012). *Methanosarcina*. The rediscovered methanogen for heavy duty biomethanation. Bioresour Technol.

[CR140] Wagner J, Coupland P, Browne HP, Lawley TD, Francis SC, Parkhill J (2016). Evaluation of PacBio sequencing for full-length bacterial 16S rRNA gene classification. BMC Microbiol.

[CR141] Wang Q, Garrity GM, Tiedje JM, Cole JR (2007). Naive Bayesian classifier for rapid assignment of rRNA sequences into the new bacterial taxonomy. Appl Environ Microbiol.

[CR142] Wang X, Li Z, Zhou X, Wang Q, Wu Y, Saino M, Bai X (2016). Study on the bio-methane yield and microbial community structure in enzyme enhanced anaerobic co-digestion of cow manure and corn straw. Bioresour Technol.

[CR143] Weiland P (2010). Biogas production. Current state and perspectives. Appl Microbiol Biotechnol.

[CR144] Witarsa F, Lansing S, Yarwood S, Gonzalez Mateu M (2016). Incubation of innovative methanogenic communities to seed anaerobic digesters. Appl Microbiol Biotechnol.

[CR145] Woese CR (1987). Bacterial evolution. Microbiol Rev.

[CR146] Xia Y, Wang Y, Fang HHP, Jin T, Zhong H, Zhang T (2014). Thermophilic microbial cellulose decomposition and methanogenesis pathways recharacterized by metatranscriptomic and metagenomic analysis. Sci Rep.

[CR147] Xia Y, Wang Y, Wang Y, Chin FYL, Zhang T (2016). Cellular adhesiveness and cellulolytic capacity in *Anaerolineae* revealed by omics-based genome interpretation. Biotechnol Biofuels.

[CR148] Yamei G, Anyi Y, Jun B, Ruxia M, Lei Y, Yanjie W, Weidong W (2017). Bioreactor performance and microbial community dynamics in a production-scale biogas plant in northeastern China. Int J Agric & Biol Eng.

[CR149] Yang D, Fan X, Shi X, Lian S, Qiao J, Guo R (2014). Metabolomics reveals stage-specific metabolic pathways of microbial communities in two-stage anaerobic fermentation of corn-stalk. Biotechnol Lett.

[CR150] Yang Y, Yu K, Xia Y, Lau FTK, Tang DTW, Fung WC, Fang HHP, Zhang T (2014). Metagenomic analysis of sludge from full-scale anaerobic digesters operated in municipal wastewater treatment plants. Appl Microbiol Biotechnol.

[CR151] Yang B, Wang Y, Qian P-Y (2016). Sensitivity and correlation of hypervariable regions in 16S rRNA genes in phylogenetic analysis. BMC Bioinformatics.

[CR152] Yarza P, Yilmaz P, Pruesse E, Glöckner FO, Ludwig W, Schleifer K-H, Whitman WB, Euzéby J, Amann R, Rosselló-Móra R (2014). Uniting the classification of cultured and uncultured bacteria and archaea using 16S rRNA gene sequences. Nat Rev Microbiol.

[CR153] Yilmaz S, Singh AK (2012). Single cell genome sequencing. Curr Opin Biotechnol.

[CR154] Yu D, Kurola JM, Lähde K, Kymäläinen M, Sinkkonen A, Romantschuk M (2014). Biogas production and methanogenic archaeal community in mesophilic and thermophilic anaerobic co-digestion processes. J Environ Manag.

[CR155] Zakrzewski M, Goesmann A, Jaenicke S, Jünemann S, Eikmeyer F, Szczepanowski R, Al-Soud WA, Sørensen S, Pühler A, Schlüter A (2012). Profiling of the metabolically active community from a production-scale biogas plant by means of high-throughput metatranscriptome sequencing. J Biotechnol.

[CR156] Zhang Q, Hu J, Lee D-J (2016). Biogas from anaerobic digestion processes. Research updates. Renew Energy.

[CR157] Zhu C, Zhang J, Tang Y, Zhengkai X, Song R (2011). Diversity of methanogenic archaea in a biogas reactor fed with swine feces as the mono-substrate by mcrA analysis. Microbiol Res.

[CR158] Zhu N, Yang J, Ji L, Liu J, Yang Y, Yuan H (2016). Metagenomic and metaproteomic analyses of a corn stover-adapted microbial consortium EMSD5 reveal its taxonomic and enzymatic basis for degrading lignocellulose. Biotechnol Biofuels.

[CR159] Ziganshin AM, Liebetrau J, Pröter J, Kleinsteuber S (2013). Microbial community structure and dynamics during anaerobic digestion of various agricultural waste materials. Appl Microbiol Biotechnol.

[CR160] Ziganshina EE, Ibragimov EM, Vankov PY, Miluykov VA, Ziganshin AM (2017). Comparison of anaerobic digestion strategies of nitrogen-rich substrates. Performance of anaerobic reactors and microbial community diversity. Waste Manag.

[CR161] Zinder SH (1990). Conversion of acetic acid to methane by thermophiles. FEMS Microbiol Lett.

