# Supplementary material for: Metagenome, metatranscriptome, and metaproteome approaches unraveled compositions and functional relationships of microbial communities residing in biogas plants
Source: Appl Microbiol Biotechnol. 2018 Apr 30;102(12):5045–63. doi: 10.1007/s00253-018-8976-7 (PMC5959977; doi:10.1007/s00253-018-8976-7)
Supplement: Supplementary file 1 — (PDF 242 kb) [file 253_2018_8976_MOESM1_ESM.pdf]

Applied Microbiology and Biotechnology

Metagenome, metatranscriptome and metaproteome approaches unraveled compositions and functional relationships of microbial communities residing in biogas plants

Julia Hassa[1‡], Irena Maus[1‡], Sandra Off[2], Alfred Pühler[1], Paul Scherer[2], Michael Klocke[3†], Andreas Schlüter[1†*]

[1] Center for Biotechnology (CeBiTec), Bielefeld University, Genome Research of Industrial Microorganisms, Universitätsstrasse 27, 33615 Bielefeld, Germany

[2] Dept. Biotechnologie, Hochschule für angewandte Wissenschaften (HAW) Hamburg Ulmenliet 20, 21033 Hamburg, Germany

[3] Dept. Bioengineering, Leibniz Institute for Agricultural Engineering and Bioeconomy, Max-Eyth-Allee 100, 14469 Potsdam, Germany

[‡ †] These authors contributed equally to this article.

[*] **Corresponding Author**

Dr. Andreas Schlüter, Center for Biotechnology (CeBiTec), Bielefeld University, Genome Research of Industrial Microorganisms, 33615 Bielefeld, Germany

Phone: +49 (0)521 106 8757

E-Mail: aschluet@CeBiTec.Uni-Bielefeld.DE

Supplemental Table S1: Summary of predominant methanogenic genera of the archaeal community and, if available, their percentage in full-scale biogas plants (BGPs) with regard to substrates, process temperatures, organic loading rates (OLRs), hydraulic retention times (HRTs), and ammonia content. Only recent publications from the years 2008 until 2017 were evaluated.

Type of BGP/substrate (partly in %)	Temp. (°C)	OLR (kg VS m^{-3} d^{-1})	HRT (d)	Ammonia (NH_4^+-N) (mg L^{-1})*	Dominant Methanogens (genus level)†	Reference‡
FRW	35	n.d.	7-10	n.d.	*Methanosaeta* (81→96%)	Lee et al. 2014
Sewage sludge	36	n.d.	22	1240	*Methanosaeta* (59%)	Luo et al. 2016
Sewage sludge	35-37	2.8	16	n.d.	*Methanosaeta* (83%)	Sundberg et al. 2013
Sewage sludge	35-37	2.1	10	250	*Methanosaeta* (69%)	Sundberg et al. 2013
FIW (62%), sewage sludge (38%)	37	2.7	28	n.d.	*Methanosaeta* (84%)	Sundberg et al. 2013
Sludge from FIW, sewage sludge	37	1.1	28	n.d.	*Methanosaeta* (87%)	Sundberg et al. 2013
Sewage sludge	36-38	2.4	17	n.d.	*Methanosaeta* (70%)	Sundberg et al. 2013
CM (46%), silage (36%), food waste (18%)	37-38	2.8	57	640→800	*Methanosaeta* (92→77%)	Franke-Whittle et al. 2014
Sewage sludge	37	n.d.	24	470	*Methanosaeta* (23%), unclassified (36%)	Luo et al. 2016
Sewage sludge	37	n.d.	30	920	*Methanosaeta* (46%)	Luo et al. 2016
Sewage sludge	37	n.d.	19	1100	*Methanosaeta* (41%)	Luo et al. 2016
Sewage sludge	MT	1.8	21	n.d.	*Methanosaeta* (~70%)	Abendroth et al. 2015
Sewage sludge	MT	1.0	29	n.d.	*Methanosaeta* (~80%)	Abendroth et al. 2015
Sewage sludge	39	n.d.	25	530	*Methanosaeta* (48%)	Luo et al. 2016
CM (76%), MS (13%), GS (5), CD (4%), grains (2%)	39	2.4	47	1640	*Methanosaeta*	Nettmann et al. 2010
CM, energy crops, agricultural by-products	43	2.5	92	1350	*Methanosaeta* (~40-60%)	Fontana et al. 2016
Sewage sludge	51-53	2.9	11	980	*Methanosaeta* (68%)	Sundberg et al. 2013

Type of BGP/substrate (partly in %)	Temp. (°C)	OLR (kg VS m^{-3} d^{-1})	HRT (d)	Ammonia (NH_4^+-N) (mg L^{-1})*	Dominant Methanogens (genus level)†	Reference‡
SHW (54%), PM/CM (33%), OFMSW (10%)	37	3.1	25	4000	*Methanobrevibacter* (96%)	Sundberg et al. 2013
OFMSW (59%), FIW (21%), PM (9%)	37	3.2	27	3400	*Methanobrevibacter* (98%)	Sundberg et al. 2013
CM/PM	37	n.d.	21	2470	*Methanobrevibacter* (62%)	Luo et al. 2016
Sewage sludge	38	n.d.	45-55	700	*Methanobrevibacter* (98%)	Sundberg et al. 2013
PM/CM (69%), SHW/OFMSW (30%)	38	3.1	29	3400	*Methanobrevibacter* (93%)	Sundberg et al. 2013
PM	39	n.d.	150	n.d.	*Methanobrevibacter* (58%)	Zhu et al. 2011
CM	39	2.2	32	1930	*Methanobrevibacter* (~20-60%), *Methanobacterium* (~20-40%)	Fontana et al. 2016
CM/PM	40	n.d.	24	2630	*Methanobrevibacter* (34%)	Luo et al. 2016
CM	42	1.6	44	1850	*Methanobrevibacter* (~30-50%), *Methanobacterium* (~30-40%)	Fontana et al. 2016
SHW (51%), CM (32%), whey (15%)	51-53	2.9	20	3900	*Methanobrevibacter* (100%)	Sundberg et al. 2013
OFMSW	35	n.d.	100	n.d.	*Methanoculleus* (>90%)	Cardinali-Rezende et al. 2012
CM mainly, fish oil waste	36	n.d.	30	n.d.	*Methanoculleus* (49%)	St-Pierre et al. 2013
OFMSW (70%), silage (20%), fat (10%)	37-40	4.0	16	2300	*Methanoculleus* (98%)	Sundberg et al. 2013
FRW	35-37	n.d.	30	n.d.	*Methanoculleus* (63→97%)	Lee et al. 2014
Silage, farm manure, livestock farming waste	MT	3.0	87	n.d.	*Methanoculleus* (59-76%)	Abendroth et al. 2015
SHW mainly	38	3.7	55	5400	*Methanoculleus* (100%)	Sundberg et al. 2013

Type of BGP/substrate (partly in %)	Temp. (°C)	OLR (kg VS m^{-3} d^{-1})	HRT (d)	Ammonia (NH_4^+-N) (mg L^{-1})*	Dominant Methanogens (genus level)†	Reference‡
PM (57%), MS (40%)	39	3.9	48	1420	*Methanoculleus*	Nettmann et al. 2010
CM (72%), MS (28%)	40	2.9	54	n.d.	*Methanoculleus* (79%)	Nettmann et al. 2008
CM/PM, industrial organic wastes	40	n.d.	32	4220	*Methanoculleus* (86%)	Luo et al. 2016
MS (63%), GR (35%), ChM (2%)	41	n.d.	40-60	n.d.	*Methanoculleus* (88%)	Schlüter et al. 2008
CM (64%), MS (37%), PM (6%), TD (2%)	41	4.0	34	3620	*Methanoculleus*	Nettmann et al. 2010
PM (50%), MS (39%), TD (9%)	44	2.5	35	3030	*Methanoculleus*	Nettmann et al. 2010
MS (82%), barley grain (12%), water (6%)	45	3.4	108	2230	*Methanoculleus*	Nettmann et al. 2010
CM/PM, industrial organic wastes	50	n.d.	11	2960	*Methanoculleus* (75%)	Luo et al. 2016
CM/PM, industrial organic wastes	52	n.d.	11	3300	*Methanoculleus* (72%)	Luo et al. 2016
CM/PM, industrial organic wastes	52	n.d.	15	2460	*Methanoculleus* (82%)	Luo et al. 2016
CM/PM, industrial organic wastes	52	n.d.	3	2310	*Methanoculleus* (80%)	Luo et al. 2016
CM/PM, industrial organic wastes	53	n.d.	11	2380	*Methanoculleus* (50%)	Luo et al. 2016
MS (56%), PM (32%), barley (6%), CM (6%)	54	8.0	20	2870	*Methanoculleus* (60%)	Maus et al. 2016
FRW	55	n.d.	39	n.d.	*Methanoculleus* (96→92%)	Lee et al. 2014
OFMSW (95%), fat (5%)	52-55	2.8	20	1800	*Methanoculleus* (46%), *Methanobacterium* (44%)	Sundberg et al. 2013
MS (97%), diverse plant materials (3%)	38	3.5	80	1330	*Methanobacterium* (43%), *Methanosaeta* (31%)	Lucas et al. 2015
CM, energy crops, agricultural by-products	50	3.2	67	2220	*Methanobacterium* (~30-60%)	Fontana et al. 2016

**Type of BGP/substrate (partly in %)**	**Temp. (°C)**	**OLR (kg VS m^{-3} d^{-1})**	**HRT (d)**	**Ammonia (NH_4^+-N) (mg L^{-1})***	**Dominant Methanogens (genus level)†**	**Reference‡**
OFMSW (34%), SHW (29%), dry fodder (16%)	52-55	3.2	20	1900	*Methanobacterium* (90%)	Sundberg et al. 2013
OFMSW (85%), SHW (15%)	55	2.1	n.d.	1700	*Methanobacterium* (56%), *Methanothermobacter* (32%)	Sundberg et al. 2013
CM (52%), food waste (48%)	55	5.2	26	3840 → 2960	*Methanothermobacter* (98→100%)	Franke-Whittle et al. 2014
FRW	55-62	n.d.	16-18	n.d.	*Methanoculleus* (100%→1%), *Methanothermobacter* (0%→99%)	Lee et al. 2014
OFMSW	MT	0.9	33	n.d.	*Methanosarcina* (~50-90%)	Abendroth et al. 2015
CM, Silage, straw	MT	1.3	26-29	n.d.	*Methanosarcina* (~40%)	Abendroth et al. 2015
CM, straw, feed residues	MT	2.1	32-35	n.d.	*Methanosarcina* (~80-95%)	Abendroth et al. 2015
CM mainly, whey	38	n.d.	21	n.d.	*Methanosarcina* (100%)	St-Pierre et al. 2013
CM mainly, ice cream factory waste	38	n.d.	25-27	n.d.	*Methanosarcina* (99%)	St-Pierre et al. 2013
MS (46-54%), CD (15%), CM (22%), Rye silage (9-14%)	41-45	1.5-1.9	120-138	2300-2700	*Methanosarcina* (~60-80%)	Theuerl et al. 2015
CM, energy crops	44	2.6	48	1820	*Methanosarcina* (~30-40%)	Fontana et al. 2016
CM, energy crops, agro-industrial by-products	45	3.0	66	2420	*Methanosarcina* (~50-60%)	Fontana et al. 2016
CM/PM, industrial organic wastes	52	n.d.	22	2530	*Methanosarcina* (90%)	Luo et al. 2016
Municipal and industrial sewage sludge (biodiesel waste)	MT	0.5	25	n.d.	*Methano-methylovorans* (40-55%), *Methanosaeta* (~40-55%)	Abendroth et al. 2015

Type of BGP/substrate	Temp. (°C)	OLR (kg VS m^{-3} d^{-1})	HRT (d)	Ammonia (NH_4^+-N) (mg L^{-1})	In order or families grouped methanogenic genera	Reference
Sewage sludge	35	n.d.	n.d.	1210	*Methanosaetaceae*	Fotidis et al. 2013
CM, PM	37	n.d.	n.d.	4570	*Methanobacteriales*	Fotidis et al. 2013
PM	38	n.d.	n.d.	2930	*Methanobacteriales*	Fotidis et al. 2013
CM, PM	52	n.d.	n.d.	2040	*Methanomicrobiales*	Fotidis et al. 2013
CM, PM	53	n.d.	n.d.	2260	*Methanosarcinaceae*	Fotidis et al. 2013
CM, PM, ChM	53	n.d.	n.d.	2440	*Methanomicrobiales*	Fotidis et al. 2013
CM, CD	55	n.d.	n.d.	2030	*Methanomicrobiales*	Fotidis et al. 2013
Sewage sludge	55	n.d.	n.d.	900	*Methanosaetaceae*	Fotidis et al. 2013
FRW	MT	n.d.	25-27	n.d.	*Methanobacteriaceae*	Han et al. 2017
FRW	MT	n.d.	30-36	n.d.	*Methanomicrobiaceae*	Han et al. 2017
Diluted food waste	MT	n.d.	21-25	n.d.	*Methanomicrobiaceae, Methanobacteriaceae*	Han et al. 2017
Food waste	MT	n.d.	30-40	n.d.	*Methanomicrobiaceae*	Han et al. 2017
Food waste	MT	n.d.	30-40	n.d.	*Methanomicrobiaceae*	Han et al. 2017
Food waste	MT	n.d.	30-40	n.d.	*Methanomicrobiaceae*	Han et al. 2017

* BGPs operate at pH-values of approx. pH 8; ammonia (NH_3) is in balance with ammonium according to pH and temperature. BS: beet silage; CD: cattle dung; ChM: chicken manure; CM: cattle liquid manure; FIW: food industry waste; FRW: food waste-recycling wastewater; GR: green rye; GS: grass silage; MS: Maize silage; OFMSW: organic fraction of municipal solid waste; PM: pig liquid manure; SHW: slaughterhouse waste; TD: turkey dung; WS: wheat straw; MT: mesophilic temperature; →: experimental period; n.d.: no data.

† Colors differentiate genera of methanogenic *Archaea*.

‡ References are listed in the main manuscript.
